# *In Situ* Generated Novel ^1^H MRI Reporter for β-Galactosidase Activity Detection and Visualization in Living Tumor Cells

**DOI:** 10.3389/fchem.2021.709581

**Published:** 2021-07-15

**Authors:** Shuo Gao, Lei Zhao, Zhiqiang Fan, Vikram D. Kodibagkar, Li Liu, Hanqin Wang, Hong Xu, Mingli Tu, Bifu Hu, Chuanbin Cao, Zhenjian Zhang, Jian-Xin Yu

**Affiliations:** ^1^ Center of Translational Medicine, Fifth School of Medicine/Suizhou Central Hospital, Hubei University of Medicine, Suizhou, China; ^2^ School of Biological and Health Systems Engineering, Arizona State University, Tempe, AZ, United States; ^3^ Department of Radiology, University of Texas Southwestern Medical Center at Dallas, Dallas, TX, United States; ^4^ Biomedical Research Institute, Hubei University of Medicine, Shiyan, China

**Keywords:** β-galactosidase detection, responsive Fe-based ^1^H-MRI agent, *T*_1_ and *T*_2_ relaxation mapping, *in vitro*^1^H-MRI imaging, *lacZ* gene reporter, synthesis

## Abstract

For wide applications of the *lacZ* gene in cellular/molecular biology, small animal investigations, and clinical assessments, the improvement of noninvasive imaging approaches to precisely assay gene expression has garnered much attention. In this study, we investigate a novel molecular platform in which alizarin 2-*O*-β-*d*-galactopyranoside **AZ-1** acts as a *lacZ* gene/β-gal responsive ^1^H-MRI probe to induce significant ^1^H-MRI contrast changes in relaxation times *T*_1_ and *T*_2_ *in situ* as a concerted effect for the discovery of β-gal activity with the exposure of Fe^3+^. We also demonstrate the capability of this strategy for detecting β-gal activity with *lacZ*-transfected human MCF7 breast and PC3 prostate cancer cells by reaction-enhanced ^1^H-MRI *T*_1_ and *T*_2_ relaxation mapping.

## Introduction

Due to various advantages such as stability, high turnover rate, and ease of conjugation, the *lacZ* gene-encoding β-galactosidase (β-gal) has been broadly used in cellular/molecular biology, small animal studies, clinical trials with assays of clonal insertion, transcriptional activation, and protein expression and interaction (Kruger et al., 1999; Haberkorn et al., 2005; Razgulin et al., 2011; Yang et al., 2019). Moreover, overexpressed β-gal has been identified as a vital enzyme biomarker related to cell senescence and cancer progression (Chatterjee et al., 1979; Alam et al., 1990; Dimri et al., 1995; Paradis et al., 2001; Pacheco-Rivera et al., 2016; Lozano-Torres et al., 2017; Sharma and Leblanc, 2017; Kim et al., 2018; Wang et al., 2019; Li et al., 2020b; Gao et al., 2020; Qiu et al., 2020). Thus, β-gal activity detection has been exploited with diverse techniques including colorimetric assays (James et al., 2000; Browne et al., 2010; Zeng et al., 2012; Yeung et al., 2013; Chen et al., 2016; Hu Q. et al., 2017), fluorescence (Tung et al., 2004; Urano et al., 2005; Josserand et al., 2007; Kamiya et al., 2007; Feng et al., 2009; Koide et al., 2009; Kamiya et al., 2011; Oushiki et al., 2012; Han et al., 2013; Sakabe et al., 2013; Lee et al., 2014; Asanuma et al., 2015; Peng et al., 2015; Zeng et al., 2015; Doura et al., 2016; Gu et al., 2016; Zhang C. et al., 2016; Zhang X. X. et al., 2016; Huang J. et al., 2017; Hu Q. et al., 2017; Jiang et al., 2017; Kim et al., 2017; Nakamura et al., 2017; Wei et al., 2017; Zhang et al., 2017; Tang et al., 2017; Chen et al., 2018; Ito et al., 2018; Liu et al., 2018; Yang et al., 2018; Chen et al., 2019; Fu et al., 2019; Gu et al., 2019; Jiang et al., 2019; Kong et al., 2019; Lee et al., 2019; Shi et al., 2019; Singh et al., 2019; Zhang et al., 2019a; Zhang X. et al., 2019; Zhao et al., 2019; Li et al., 2020a; Li Y. et al., 2020; Li Z. et al., 2020; Pang et al., 2020; Wu et al., 2020; Zhu et al., 2020), chemiluminescence (Wehrman et al., 2006; Liu and Mason, 2010; Broome et al., 2015; Green et al., 2017; Huang Y. et al., 2017; Wang et al., 2017; Gorai and Maitra, 2018; Hananya and Shabat, 2019; Lozano-Torres et al., 2019; Zhang et al., 2019b), positron emission tomography or single-photon emission computed tomography (Celen et al., 2008; Van Dort et al., 2008; Rempel et al., 2017), magnetic resonance imaging (MRI) (Louie et al., 2000; Chang et al., 2007; Hanaoka et al., 2008; Cui et al., 2010; Bengtsson et al., 2010; Arena et al., 2011; Yu et al., 2012a; Gulaka et al., 2013; Li et al., 2013; Heffern et al., 2014; Burke et al., 2015; Hingorani et al., 2015; Fernández-Cuervo et al., 2016; Hu J. et al., 2017; Li and Meade, 2019; Xu et al., 2019; Lilley et al., 2020), and ^19^F-MRS/MRI approaches (Yu et al., 2005; Kodibagkar et al., 2006; Yu et al., 2006; Yu and Mason, 2006; Liu et al., 2007; Yu et al., 2008a; Yu et al., 2008b; Mizukami et al., 2011; Yu et al., 2012b; Yu et al., 2013; Yu et al., 2017). In particular, ^1^H-MRI molecular imaging approaches for visualization of β-gal activity attract much more attention because ^1^H-MRI is noninvasive and capable of soft tissue delineation with a high lateral and depth resolution (Terreno et al., 2010; Haris et al., 2015; Wahsner et al., 2019).

**FIGURE 1:**
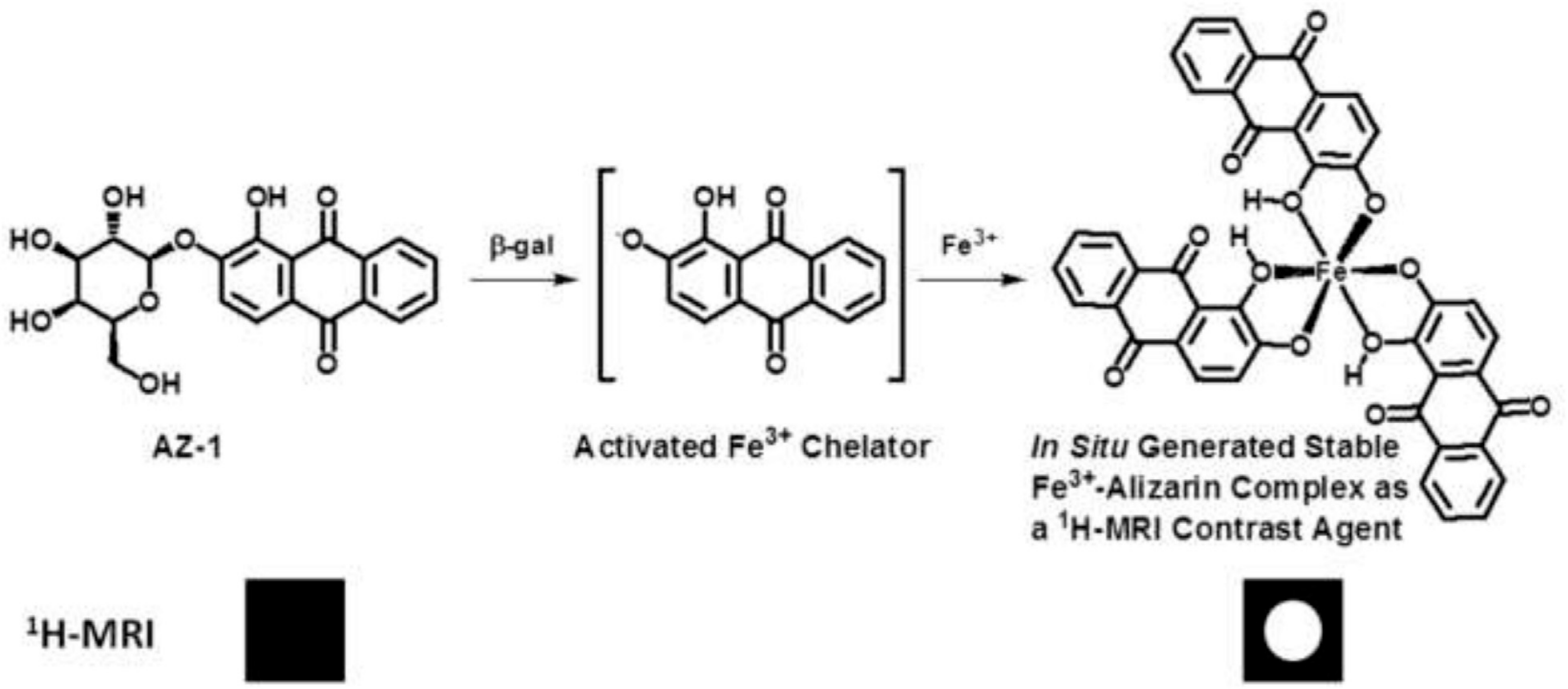
The proposed mechanism of an *in situ* generated stable Fe^3+^–alizarin complex for ^1^H MRI detection of β-gal activity.

β-Galactosidase prompts the hydrolysis of β-*d*-galactopyranoside by cleavage of its β-anomeric C-O linkage between β-*d*-galactopyranose and aglycone; the hydrolysis reactivity of β-*d*-galactopyranosides to β-gal is completely dependent upon the aglycone structure. However, the structure activity relationship of the aglycones in β-*d*-galactopyranosides vs. β-gal is not yet clear (Juers et al., 2012; Duo et al., 2017). Therefore, further exploration is still highly needed to discover effective β-gal substrates for functional molecular imaging probes. We believe that the traditional histopathological methods of assaying β-gal activity might be the fruitful resources for developing novel imaging agents for the assessment of *lacZ* gene expression. In reviewing the histopathological literature, we noticed that the well-established β-gal substrate alizarin 2-*O*-β-*d*-galactopyranoside **AZ-1** (Figure 1) is readily hydrolyzed by β-gal to release aglycone alizarin, which chelates with ferric iron Fe^3+^ to form an intense dark violet Fe complex (James et al., 2000). By comparison of the structural characteristics of the Fe^3+^–alizarin complex with Fe^3+^-based ^1^H-MRI contrast agents (Davies et al., 1996; Richardson et al., 1999; Schwert et al., 2002; Schwert et al., 2005; Haas and Franz, 2009; Yu et al., 2012a; Yu et al., 2012b; Gulaka et al., 2013; Li et al., 2013; Yu et al., 2013; Kuznik and Wyskocka, 2016), we speculated that the Fe^3+^–alizarin complex could function as an Fe^3+^-based ^1^H-MRI contrast agent. If so, the well-established β-gal substrate **AZ-1** could work as a *lacZ* gene or β-gal ^1^H-MRI reporter. Upon delivery and cleavage at *lacZ*-transfected or β-gal–overexpressed tumor cells with the presence of Fe^3+^, the paramagnetic Fe complex could be spontaneously formed *in situ* and specifically produced the ^1^H MRI contrast effect while localizing and accumulating ^1^H-MRI signals at the β-gal activity site. Figure 1 depicts the Fe^3+^–alizarin complex generated *in situ* for the ^1^H-MRI detection of β-gal activity. We now demonstrate the use of exploiting **AZ-1** to assess β-gal activity *in vitro* with *lacZ*-transfected human MCF7 breast and PC3 prostate cancer cells by ^1^H MRI *T*_*1*_ and *T*_*2*_ relaxation mapping.

## Results and Discussion

### Verification of the Fe^3+^–Alizarin Complex as an ^1^H-MRI Contrast Agent

**FIGURE 2:**
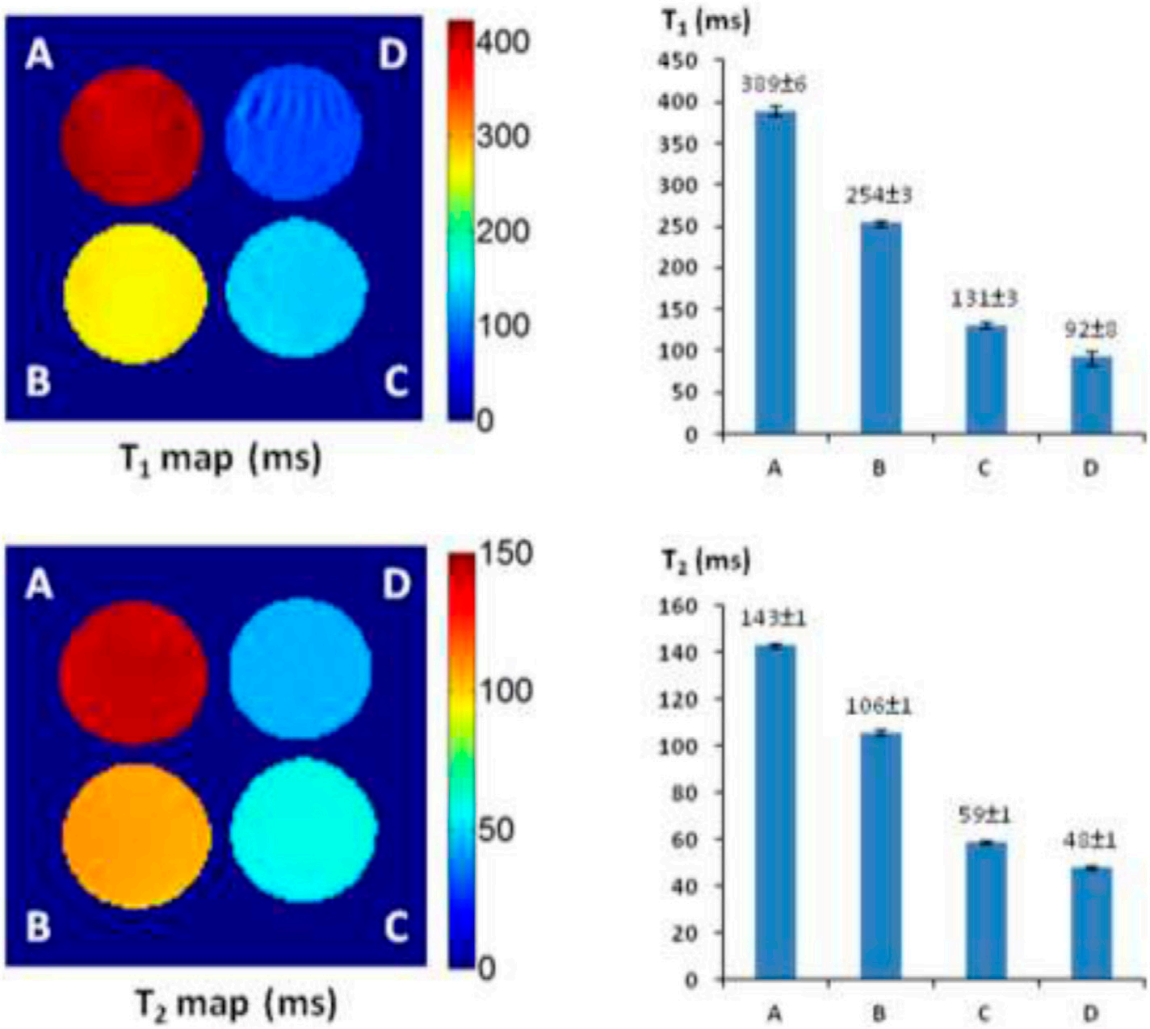
The ^1^H-MRI of an Fe^3+^–alizarin complex. MRI acquisition parameters: ^1^H-MRI, 200 MHz, matrix size: 128 × 128, FOV: 40 mm × 40 mm, slice thickness: 2 mm; receiver bandwidth: 20 kHz, *T*_1_-map: saturation recovery spin-echo sequence, TR = 200, 400, 600, 800, 1,000, 2000, 3,000, and 6,000 ms, respectively, TE = 15 ms; *T*_2_-map: multi-echo SE sequence, TE = 10, 20, 30, 40, 50, 60, 70, 80, 90, 100, 110, 120, 130, 140, 150, and 160 ms, respectively, TR = 2000 ms. **(A)** Control, FAC (15.0 mM); (**B**) alizarin (2.5 mM), FAC (15.0 mM); **(C)** alizarin (6.0 mM) and FAC (15.0 mM); **(D)** alizarin (9.0 mM), FAC (15.0 mM) in 1:1 (V/V′), and DMSO/PBS (0.1 M, pH = 7.4) at 37°C in 4 h.

Alizarin is 1,2-dihydroxy-9,10-anthraquinone with a tricyclic aromatic planar structure and chelates with Fe^3+^ to form a thermodynamically stable octahedral Fe^3+^–alizarin (1:3) complex at physiological pH conditions with the stability constant logβ = 32.21 (Das et al., 1995; Das et al., 2002). To explore the MRI signal–enhancing capability of the Fe^3+^–alizarin complex, the spin–lattice relaxation time *T*_1_ and spin–spin relaxation time *T*_2_ of the Fe^3+^–alizarin complex were measured with a 4.7 T MR scanner using a saturation recovery spin echo sequence and multi-spin echo sequence with varying repetition times (TRs) and echo times (TEs), respectively. The images were acquired using a 3-cm diameter solenoid coil (home-built) with 4 × 4 cut section of a 96-well plate containing the different concentrations of alizarin and ferric ammonium citrate (FAC) mixed solutions in 1:1 (V/V’) DMSO/PBS (0.1 M, pH = 7.4) at 37°C. Figure 2 displays the significant changes as expected on the *T*_1_ and *T*_2_ maps and relaxation time values of the Fe^3+^–alizarin complex at *T*_1_ = 254 ± 3, 131 ± 3, and 92 ± 8 ms, and *T*_2_ = 106 ± 1, 59 ± 1, and 48 ± 1 ms, corresponding to the concentrations of alizarin at 2.5, 6.0, and 9.0 mM, respectively. The comparison with the control FAC of *T*_1_ = 389 ± 6 ms and *T*_2_ = 143 ± 1 ms showed that the Fe^3+^–alizarin complex formed *in situ* resulted in substantial signal enhancement on either *T*_1_- or *T*_2_-weighted ^1^H-MRI, confirming the Fe^3+^–alizarin complex generated *in situ* to function as an ^1^H-MRI contrast agent. Notably, the significantly different *T*_1_ and *T*_2_ values of the Fe^3+^–alizarin complex suggested the potential to combine *T*_1_ and *T*_2_ data for additional information of imaging evaluation and detection reliability, specifically where there is possibility for misinterpretation in tissue heterogeneity (Zhou et al., 2017).

### Alizarin β-*d*-Galactopyranoside Synthesis

After the Fe^3+^–alizarin complex was shown to be an ^1^H-MRI contrast agent, we therefore began the β-*d*-galactopyranosylation with alizarin at the phenolic hydroxyl groups. Previously, James et al. (2000) reported a modified method for the synthesis of **AZ-1**, involving the reaction of alizarin potassium salt with acetobromo-α-*D*-galactose *via* the nucleophilic substitution procedure followed by deacetylation mediated by the aqueous NaOH solution, but the yield was low (14%). We observed that the phenolic hydroxyl groups at 1,2-positions of alizarin have excellent site-reaction selectivity due to the various electronic deficiency/sterically hindered effects (Mahal et al., 2011) and apparently different *pKa* values: *pKa*_(2-OH)_ = 5.98 ± 0.05, whereas *pKa*_(1-OH)_ = 9.88 ± 0.05 (Das et al., 1995; Das et al., 2002), which suggested that the phase-transfer catalysis method at pH = 8-9 could provide regio- and stereoselective synthesis of **AZ-1**, as exploited previously for β-gal ^19^F-MRS/MRI reporters (Yu et al., 2005; Kodibagkar et al., 2006; Yu et al., 2006; Yu and Mason, 2006; Liu et al., 2007; Yu et al., 2008a; Yu et al., 2008b; Yu et al., 2012b; Yu et al., 2013; Yu et al., 2017). To the well-stirred solution of alizarin in CH_2_Cl_2_-H_2_O (pH 8–9) using tetrabutylammonium bromide (TBAB) as a catalyst at 50°C, an equimolar amount of 2, 3, 4, and 6-tetra-*O*-acetyl-α-*D*-galactopyranosyl bromide was dropped under N_2_ atmosphere for around 1 h; alizarin 2-*O*-2′, 3′, 4′, and 6′-tetra-*O*-acetyl-β-*D*-galactopyranoside **AZ-M1** was isolated purely at the yield of 78%. The NOESY correlation between anomeric H-1′ and H-3 in **AZ-M1** (Supporting Information, Supplementary Figure S7) showed that β-*d*-galactopyranosylation took place at the 2-hydroxyl group of alizarin as predicted. The following deacetylation with NH_3_/MeOH from 0°C to room temperature produced **AZ-1** at 91% yield.

**FIGURE 3:**
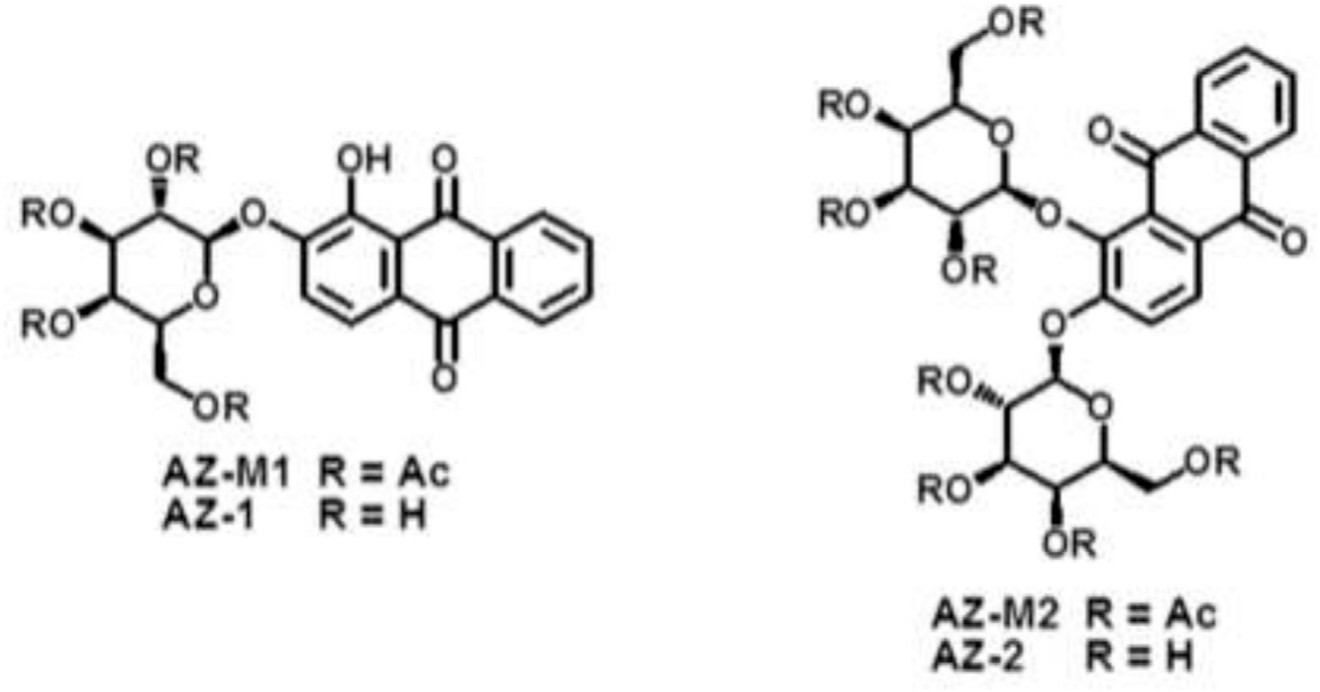
The structures of alizarin mono β-*D*-galactopyranosides **AZ-M1**/**AZ-1** , and alizarin di-β-*D*-galactopyranosides **AZ-M2**/**AZ-2**.

The prerequisite for molecular MRI of intracellular targets is that the contrast agents must be effectively taken up by cells *in vivo*, which requires contrast agents to be sufficiently soluble and capable of entering cells. Carbohydrate-associated prodrugs in clinical applications have widely demonstrated the improved aqueous solubility and permeability, leading to better selectivity and efficacy for diagnosis and therapy (Dwek, 1996; Bertozzi and Kiessling, 2001; Hudak and Bertozzi, 2014; Fernández-Tejada et al., 2015). We hence thought about introducing an additional β-*D*-galactopyranosyl unit to form alizarin 1,2-di-*O*-β-*d*-galactopyranoside **AZ-2**. Similarly, a drop of 2.2 equivalent 2, 3, 4, and 6-tetra-*O*-acetyl-α-*D*-galactopyranosyl bromide CH_2_Cl_2_ solution into alizarin in CH_2_Cl_2_-H_2_O (pH 10–11) employing TBAB as a catalyst at 55°C under N_2_ atmosphere afforded alizarin 1,2-di-*O*-2′, 3′, 4′, 6′-tetra-*O*-acetyl-β-*D*-galactopyranoside **AZ-M2** at 62% yield. Deacetylation accomplished the target molecule alizarin 1,2-di-*O*-β-*d*-galactopyranoside **AZ-2** with 94% yield. Figure 3 illustrates the structures of **AZ-1**/**AZ-M1** and **AZ-2**/**AZ-M2**. As expected, the free di-β-*d*-galactopyranoside **AZ-2** is soluble in PBS (0.1 M, pH = 7.4) at the concentration of 15 mM; meanwhile, the free mono-β-*d*-galactopyranoside **AZ-1** unlikely requires the addition of DMSO for the same concentration. The structures of **AZ-M1**/**AZ-1** and **AZ-M2**/**AZ-2** were confirmed by NMR and HRMS data. The molecular/quasimolecular ions of **AZ-M1** and **AZ-1**, as well as **AZ-M2** and **AZ-2**, showed the introduction of one and two galactopyranosyl units to **AZ-M1**/**AZ-1** and **AZ-M2**/**AZ-2**, respectively, in which the β-*D*-galactopyranoside configuration was determined by 1H/13C NMR data of the anomeric protons at δ_H-1__′_ = δ_H-1__′′_ = 4.90–5.30 ppm and their coupling constants *J*_1__′__,2__′_ = *J*_1__′′__,2__′′_≅8.0 Hz while maintaining the corresponding anomeric C-1′/C-1′′ at δ_C-1__′_ = δ_C-1__′′_ = 99.5–104.0 ppm in accordance (Supporting Information, Supplementary Figures S4–S13), which are in agreement with the typical characteristics for the identification of the anomeric β-*d*-configuration (Yu et al., 2005; Yu et al., 2006; Yu and Mason, 2006; Kodibagkar et al., 2006; Liu et al., 2007; Yu et al., 2008a; Yu et al., 2008b; Yu et al., 2012b; Yu et al., 2013; Yu et al., 2017).

### β-Gal Reactivity

**FIGURE 4:**
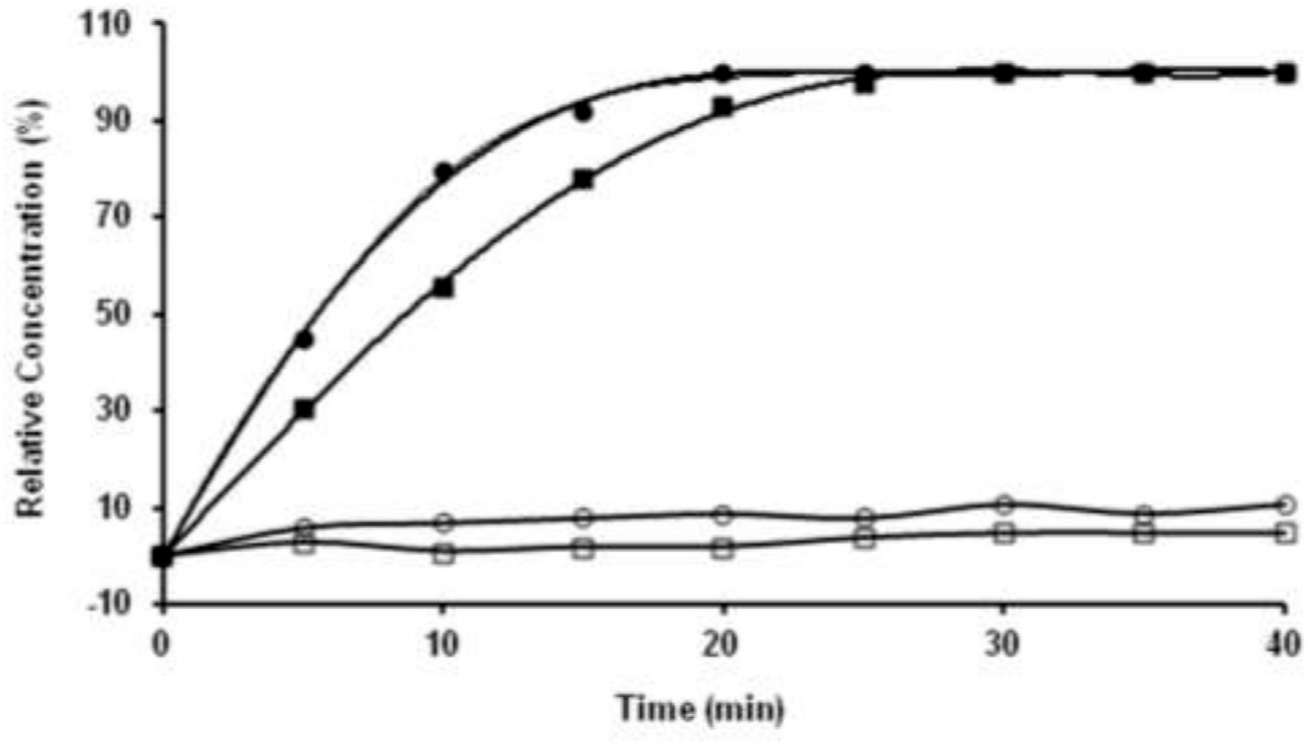
The kinetic hydrolysis time courses of alizarin β-*D*-galactopyranosides **AZ-1** (■) and **AZ-2** (●) with β-gal. Absorbance measurements at λ = 520 nm following the addition of β-gal (E801A, 3 units) to solutions of **AZ-1**, **AZ-2** each (5.0 mM) in 1:1 (V/V′) DMSO/PBS (0.1 M, pH = 7.4) at 20–22°C in different time points; The time courses of alizarin β-*D*-galactopyranosides **AZ-1** (□) and **AZ-2** (○) each (5.0 mM) in 1:1 (V/V′) DMSO/PBS (0.1 M, pH = 7.4) at 20–22°C in different time points without β-gal (E801A).

**AZ-1** has been identified as a highly sensitive substrate for the demonstration of β-gal in a range of Gram-negative bacteria under incubation at 37°C in air for 18 h (James et al., 2000). However, none of the existing data have shown the kinetics of **AZ-1** vs. β-gal, which is crucial for further *in vivo* imaging applications. The absorption spectra of **AZ-1** and **AZ-2** solutions in 1:1 (V/V′) DMSO/PBS (0.1 M, pH = 7.4) with and without β-gal (E801A) at 20–22°C indicated that upon reactions of **AZ-1** and **AZ-2** with β-gal, a new absorption around 520 nm, corresponding to the *in situ* released alizarin mono-/dianions, appeared and increased gradually. Hence, the absorbance measurements at 520 nm following the enzymatic reaction of **AZ-1** and **AZ-2** with β-gal (E801A) at 20–22°C in different time points showed that both **AZ-1** and **AZ-2** are very reactive to β-gal (E801A) with varying hydrolytic rates at ν_(AZ-1)_ = 93.3 and ν_(AZ-2)_ = 133.3 μM/min/unit, respectively (Figure 4). Also, the absorption spectra of **AZ-1** and **AZ-2** by reaction with other enzymes α-galactosidase (Sigma G7163) and β-glucuronidase (Sigma G8295) at 20–22°C; showed that both **AZ-1** and **AZ-2** remained essentially stable over the period of 60 min, verifying their specificity for reaction to β-gal.

### ^1^H-MRI Detection of β-Gal Activity

**FIGURE 5:**
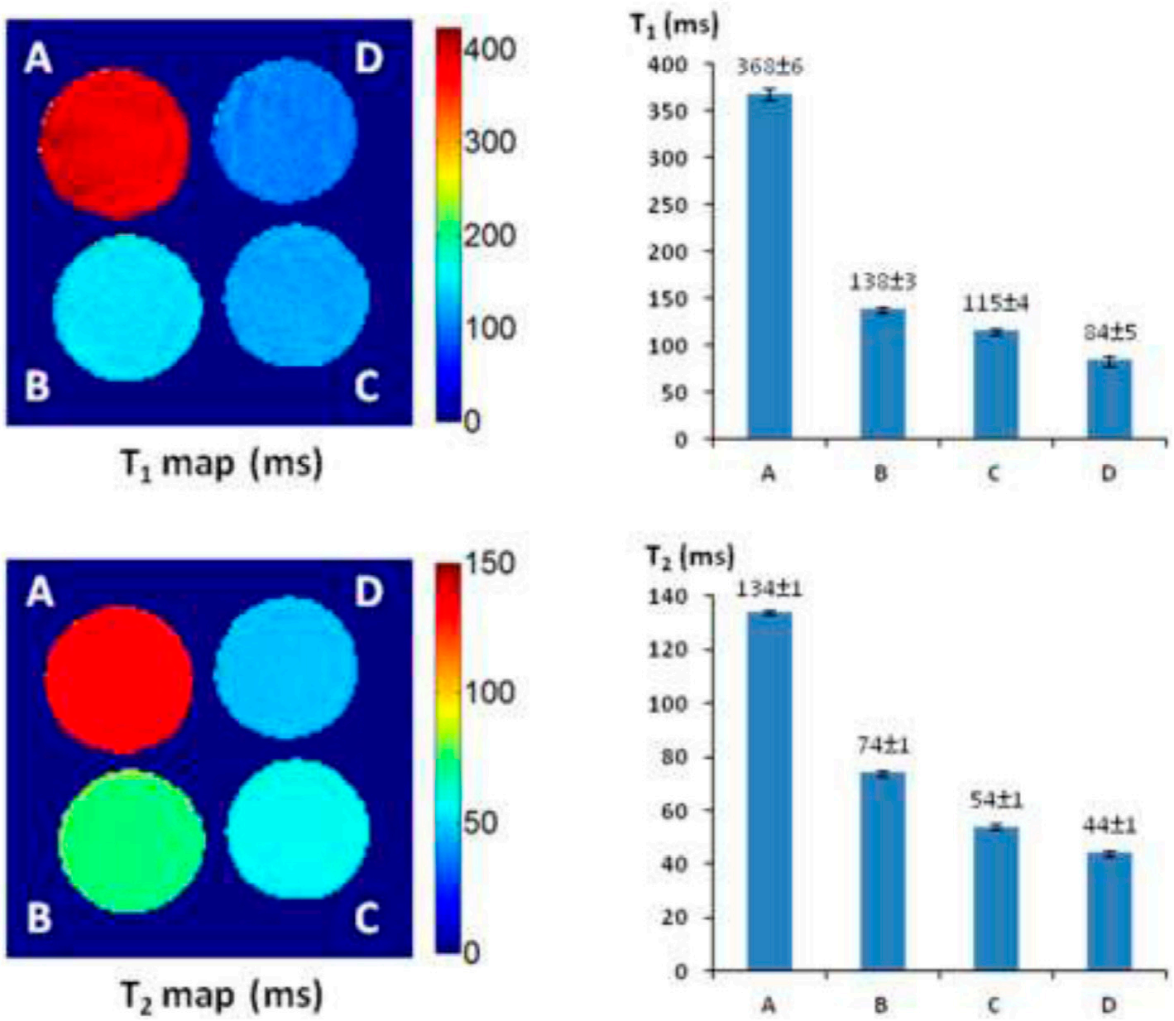
^1^H-MRI detection of β-gal activity. ^1^H-MRI acquisition: using the same parameters as in Figure 2, **(A)** alizarin 2-*O*-β-*D*-galactopyranoside **AZ-1** (9.0 mM) and FAC (15.0 mM); **(B)** alizarin 2-*O*-β-*D*-galactopyranoside **AZ-1** (4.0 mM), FAC (15.0 mM), and β-gal (E801A, 5 units); **(C)** alizarin 2-*O*-β-*D*-galactopyranoside **AZ-1** (6.0 mM), FAC (15.0 mM), and β-gal (E801A, 5 units); **(D)** alizarin 2-*O*-β-*D*-galactopyranoside **AZ-1** (9.0 mM), FAC (15.0 mM), and β-gal (E801A, 5 units) in 1:1 (V/V′) DMSO/PBS (0.1 M, pH = 7.4) at 37°C in 4 h.

**FIGURE 6:**
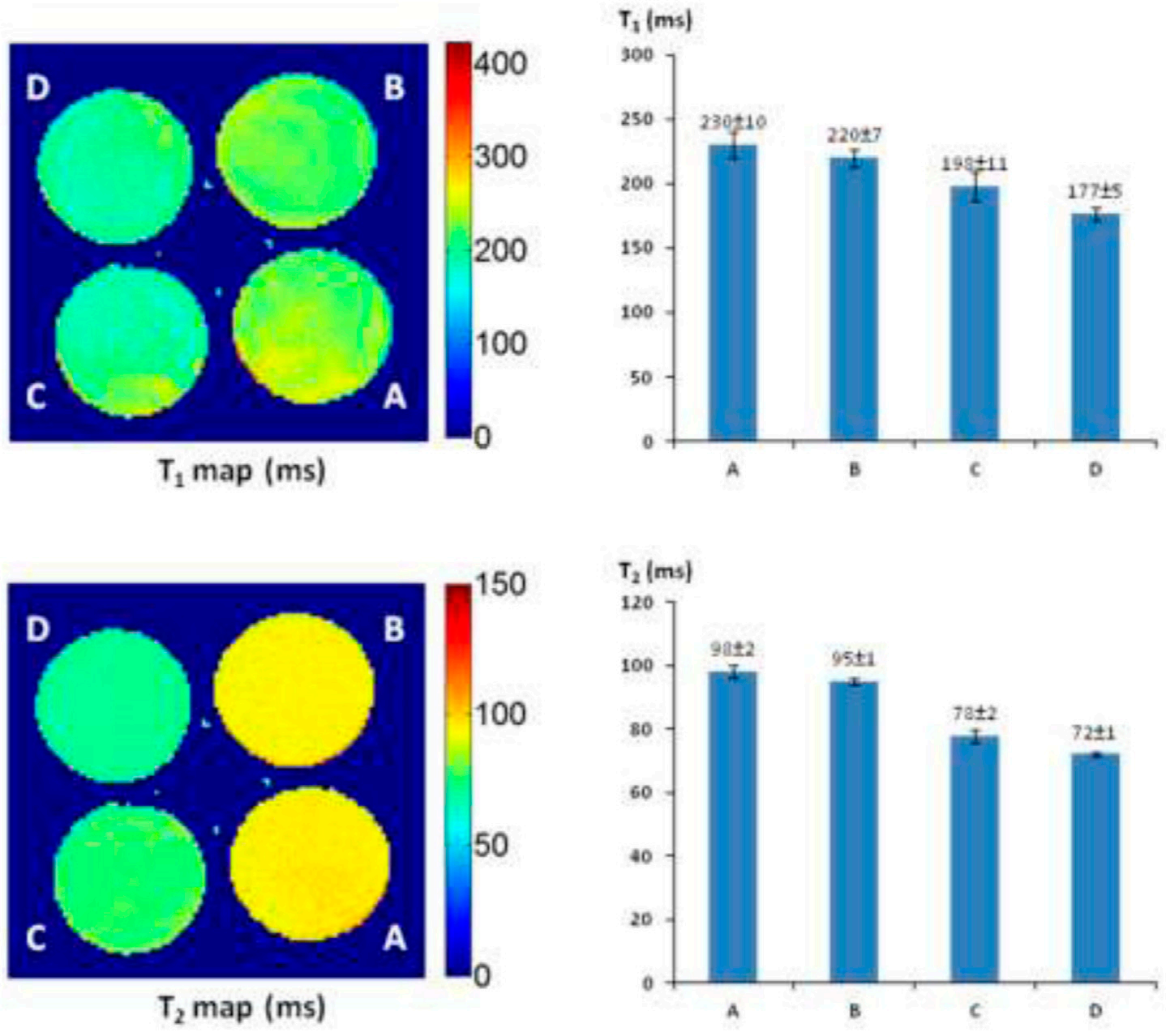
^1^H-MRI detection of β-gal activity. ^1^H-MRI acquisition: using the same parameters as in Figure 2, **(A)** alizarin 1,2-di-*O*-β-*D*-galactopyranoside **AZ-2** (9.0 mM) and FAC (15.0 mM); **(B)** alizarin 1,2-di-*O*-β-*D*-galactopyranoside **AZ-2** (4.0 mM), FAC (15.0 mM), and β-gal (E801A, 5 units); **(C)** alizarin 1,2-di-*O*-β-*D*-galactopyranoside **AZ-2** (6.0 mM), FAC (15.0 mM), and β-gal (E801A, 5 units); **(D)** alizarin 1,2-di-*O*-β-*D*-galactopyranoside **AZ-2** (9.0 mM), FAC (15.0 mM), and β-gal (E801A, 5 units) in 1:1 (V/V′) DMSO/PBS (0.1 M, pH = 7.4) at 37°C in 4 h.

The *T*_1_ and *T*_2_ maps and relaxation time values were measured with a 4 × 4 cut section of 96-well plate containing various concentrations of **AZ-1** and **AZ-2** (4.0–9.0 mM) together with a fixed concentration of FAC (15.0 mM), respectively, in 1:1 (V/V′) DMSO/PBS (0.1 M, pH = 7.4) with or without β-gal (E801A). In the **AZ-1**/FAC solution at 37°C in 4 h in the absence of β-gal, relaxation times were determined to be *T*_1_ = 368 ± 6 and *T*_2_ = 134 ± 1 ms. In comparison, in the presence of β-gal (E801A, 5 units) in the mixture solution of **AZ-1** and FAC at 37°C in 4 h, pronounced shortening in relaxation times *T*_1_ and *T*_2_ caused by the Fe^3+^–alizarin complex generated *in situ* was observed at *T*_1_ = 138 ± 3, 115 ± 4, and 84 ± 5 ms, whereas *T*_2_ = 74 ± 1, 54 ± 1, and 44 ± 5 ms, correlating with the increasing concentrations of **AZ-1** from 4.0, 6.0 and 9.0 mM, respectively (Figure 5). However, the much more soluble and reactive **AZ-2**, exhibiting significant advantages for *in vivo* ^1^H-MRI applications, produced very unexpected results under similar procedures at the same conditions. In the absence of β-gal at 37°C in 4 h, the **AZ-2**/FAC solution, as the control, yielded surprisingly reduced *T*_1_ = 230 ± 11 and *T*_2_ = 98 ± 1 ms (Figure 6). However, in the presence of β-gal (E801A, 5 units), the mixture solutions of **AZ-2**/FAC gave rise to an insignificant decrease in *T*_1_ = 220 ± 7, 198 ± 11, and 177 ± 5 ms and *T*_2_ = 95 ± 1, 78 ± 2, and 72 ± 1 ms (**AZ-2** concentrations at 4.0, 6.0, and 9.0 mM, respectively, Figure 6), indicating the much less Fe^3+^–alizarin complex formed *in situ* during the β-gal hydrolysis. Comparing the interactions between **AZ-1/AZ-2** and Fe^3+^ with their relaxivities to FAC, we attributed that the larger differences of **AZ-2**/FAC to FAC solution (*T*_1_: 230 ± 11 vs. 389 ± 6 ms and *T*_2_: 98 ± 1 vs. 143 ± 1 ms; alternatively, Δ*R*_1_ = 1.78 s^−1^ and Δ*R*_2_ = 3.21 s^−1^) than **AZ-1**/FAC solution (*T*_1_: 368 ± 6 vs. 389 ± 6 ms, *T*_2_: 134 ± 1 vs. 143 ± 1 ms; alternatively, Δ*R*_1_ = 0.15 s^−1^, Δ*R*_2_ = 0.47 s^−1^) were risen from the formation of the much stronger and more stable molecular tweezer complex **AZ-2**/Fe^3+^ due to the adjacent 3′,4′,6′-OH and 3″,4″,6″-OH located at the same side of 1,2-di-O-β-*d*-galactopyranosyl rings in the favored configuration for chelation of Fe^3+^ (Supplementary Figure S3 in the Supporting Information) (Davies et al., 1996; Richardson et al., 1999; Schwert et al., 2002; Schwert et al., 2005; Haas and Franz, 2009; Coskuner et al., 2011; Kuznik and Wyskocka, 2016) which thus simultaneously hindered the reaction with β-gal (E801A) and slowed down the release of alizarin as well as the *in situ* generation of the Fe^3+^–alizarin complex. These were confirmed by mixing solutions of **AZ-2** and β-gal (E801A) first for hydrolysis, and then followed by adding FAC for complexation at 37°C in 2 h for each step with the same concentrations as in Figure 6. A significant decrease in relaxation times *T*_1_ and *T*_2_ was seen at *T*_1_ = 133 ± 1, 110 ± 2, and 78 ± 2 ms and *T*_2_ = 73 ± 2, 51 ± 3, and 41 ± 1 ms, which were very close to expectations based on **AZ-1**/FAC *T*_1_ and *T*_2_ data as shown in Figure 5.

### *In Vitro* ^1^H-MRI Detection of *lacZ* Transfection in Human Tumor Cells

**FIGURE 7:**
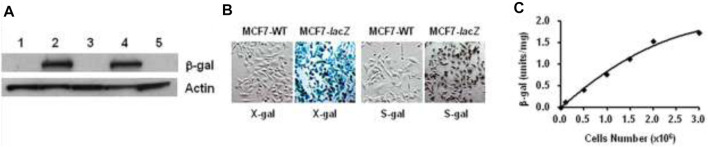
MCF7-*lacZ* transfection. **(A)** Western blot: protein extracts from two transfected MCF7-*lacZ* cell lines (lanes 2,4), together with MCF7-WT cells (lanes 1,3,5) showing intense bands for β-gal activity in MCF7-*lacZ* cells and none in MCF7-WT cells; **(B)** MCF7-WT/MCF7-*lacZ* cells staining by X-gal and S-gal: deep blue (X-gal) and black (S-gal) staining confirming the intense *lacZ* expression in MCF7-*lacZ* cells with essentially no β-gal activity in MCF7-WT cells. Regional magnification ×100; **(C)** β-Gal assay for activity quantification in MCF7-*lacZ* cells: 1.0 unit corresponding to the hydrolysis of 1.0 μmol/min *O*-nitrophenyl β-*D*-galactopyranoside, β-gal activity was increasingly associated with the number of MCF7-*lacZ* cells.

The recombinant vector phCMV*lacZ* has been successfully created and used to stably transfect human prostate cancer PC3-*lacZ* cells from PC3-wild-type (WT) cells (Liu et al., 2007). Accordingly, human breast cancer MCF7-*lacZ* cells were stably transfected from MCF7 wild-type (WT) cells: the β-gal activity and quantification in MCF7-*lacZ* cells were verified on the basis of Western blot, X-gal and S-gal staining, and the β-gal assay (Figure 7).

**FIGURE 8:**
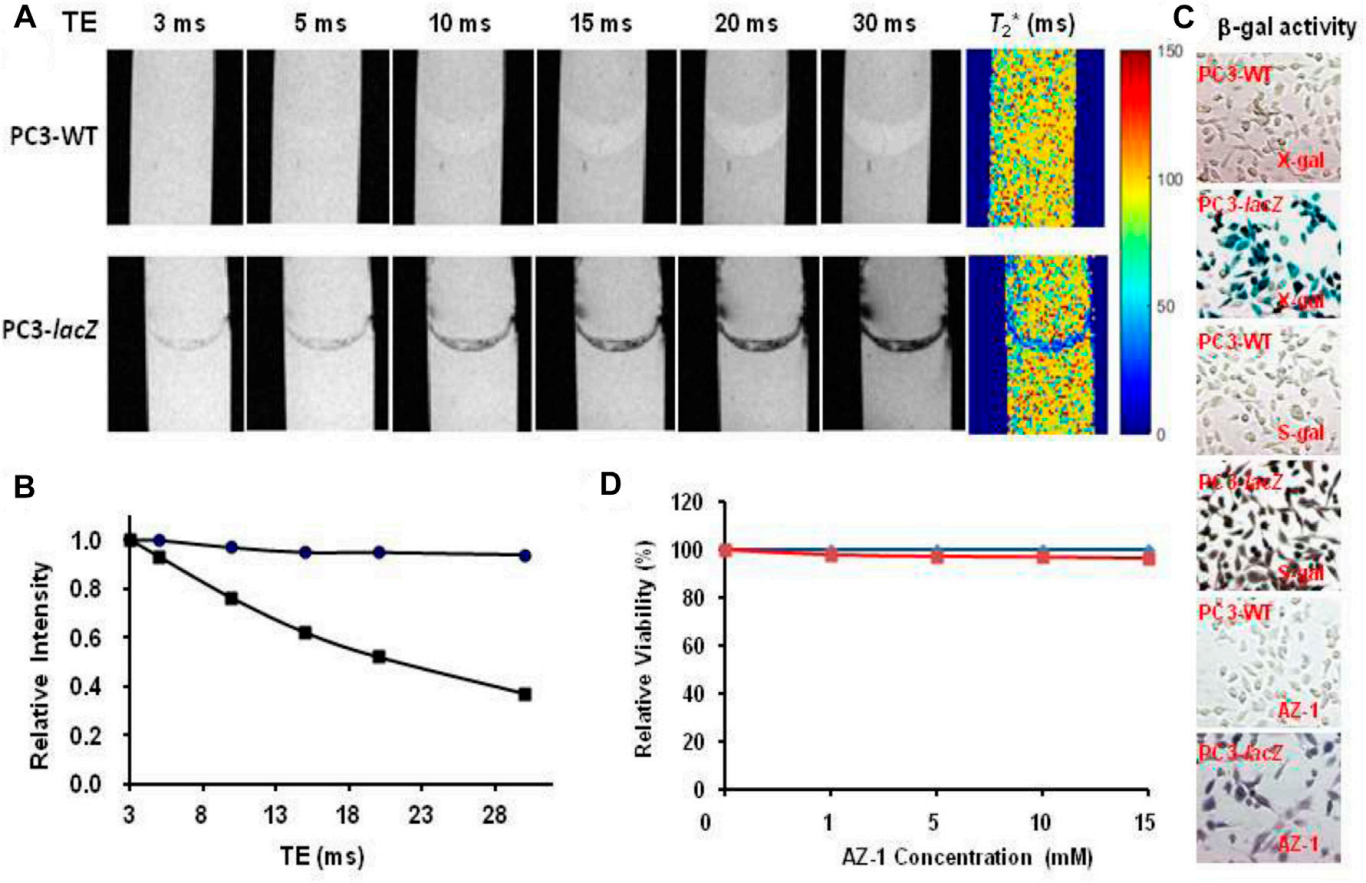
*In vitro* ^1^H-MRI detection of *lacZ* gene expression in PC3-*lacZ* cells. ^1^H-MRI acquisition: ^1^H-MRI, 400 MHz, matrix size: 256 × 128, FOV: 48 mm × 24 mm, gradient echo imaging: TE = multiple values 3–30 ms, TR = 100 ms, flip angle = 10° **(A)** *T*_2_* maps: A mixture of alizarin 2-*O*-β-*D*-galactopyranosides **AZ-1** (10.0 mM) and FAC (10.0 mM) with 5 × 10^5^ PC3-WT or PC3-*lacZ* cells was placed in the interlayer between 1% low-gelling temperature agarose in a 10-mm NMR tube, and then incubated for 4 h at 37°C under 5% CO_2_/air with 95% humidity, *T*_2_*_(**AZ-1/PC3-WT/FAC**)_ = 96 ± 23 ms (top row) and *T*_2_*_(**AZ-1/PC3-*lacZ*/FAC**)_ = 26 ± 14 ms (bottom row), respectively; **(B)** relative signal intensity changes at different echo times (TEs) from (**A**), essentially no signal loss with PC3-WT cells (●) but a significant signal loss with PC3-*lacZ* cells (■); **(C)** PC3-WT/PC3-*lacZ* cells staining by X-gal, S-gal, and **AZ-1**: deep blue (X-gal), black (S-gal), and dark violet (**AZ-1**) staining confirming an intense *lacZ* expression in PC3-*lacZ* cells with essentially no β-gal activity in PC3-WT cells. Regional magnification ×100; **(D)** cytotoxicity: PC3-WT/PC3-*lacZ* cells viability *vs.* **AZ-1** in various concentrations in 1:1 (V/V′) DMSO/PBS (0.1 M, pH = 7.4) at 37°C under 5% CO_2_/air with 95% humidity for 72 h, PC3-WT cells (in blue), and PC3-*lacZ* cells (in red)).

Given **AZ-2** showed much better aqueous solubility and reactivity to β-gal, the stabilized molecular tweezer complexation **AZ-2**/Fe^3+^ obstructed its implementation spreading to effective ^1^H-MRI assessment of β-gal. So, **AZ-1** with a significant signal loss either on *T*_1_ or *T*_2_ upon β-gal hydrolysis was prompted for the further *in vitro* ^1^H-MRI evaluation. As an initial demonstration for *in vitro* ^1^H-MRI detection of β-gal with *lacZ*-transfected human cancer cells, we first acquired *T*_2_^*^ maps on pair mixtures of **AZ-1** (10.0 mM) with PC3-WT cells (5 × 10^5^) and PC3-*lacZ* cells (5 × 10^5^), respectively, in the presence of FAC (10.0 mM) layered between agarose after incubation 4 h at 37°C under 5% CO_2_/air with 95% humidity. Significant differences confined within the layers were observed between PC3-WT and PC3-*lacZ* cells at different echo times (Figure 8A), in which there was essentially no signal loss with PC3-WT cells but a remarkable signal decrease with PC3-*lacZ* cells upon increasing echo times (TEs) (Figure 8B). The relaxation time *T*_2_^*^ was determined to be *T*_2_^*^_(**AZ-1/PC3/FAC**)_ = 96 ± 23 ms in PC3-WT cells, while *T*_2_^*^_(**AZ-1/PC3-*lacZ*/FAC**)_ = 26 ± 14 ms in PC3-*lacZ* cells. Again, the β-gal activity was verified based on X-gal, S-gal, and **AZ-1** staining (dark violet) (Figure 8C), with each staining method consistently showing intense *lacZ* expression in PC3-*lacZ* cells with essentially no β-gal activity in PC3-WT cells.

The cytotoxicity of **AZ-1** was studied with PC3-WT and PC3-*lacZ* cells in PBS (0.1 M, pH = 7.4) incubated 72 h at 37°C under 5% CO_2_/air with 95% humidity. Cell viability assays showed that neither **AZ-1** nor alizarin showed toxicity to PC3 cells, for **AZ-1** viability exceeded 96% for both PC3-WT and PC3-*lacZ* cells at all concentrations tested (Figure 8D).

Furthermore, *in vitro* ^1^H-MRI acquisition of **AZ-1** (10.0 mM) with PC3-WT cells (5 × 10^5^) and PC3-*lacZ* cells (5 × 10^5^) in the presence of FAC (10.0 mM) was performed in a 1:1 (V/V′) DMSO/PBS (0.1 M, pH = 7.4) solution. A pronounced signal decrease in the relaxation time *T*_1_ was observed between PC3-WT (*T*_1(**AZ-1/PC3-WT/FAC**)_ = 245 ± 9 ms) and PC3-*lacZ* cells (*T*_1(**AZ-1/PC3-*lacZ*/FAC**)_ = 82 ± 7 ms) after incubation 4 h at 37°C under 5% CO_2_/air with 95% humidity.

**FIGURE 9:**
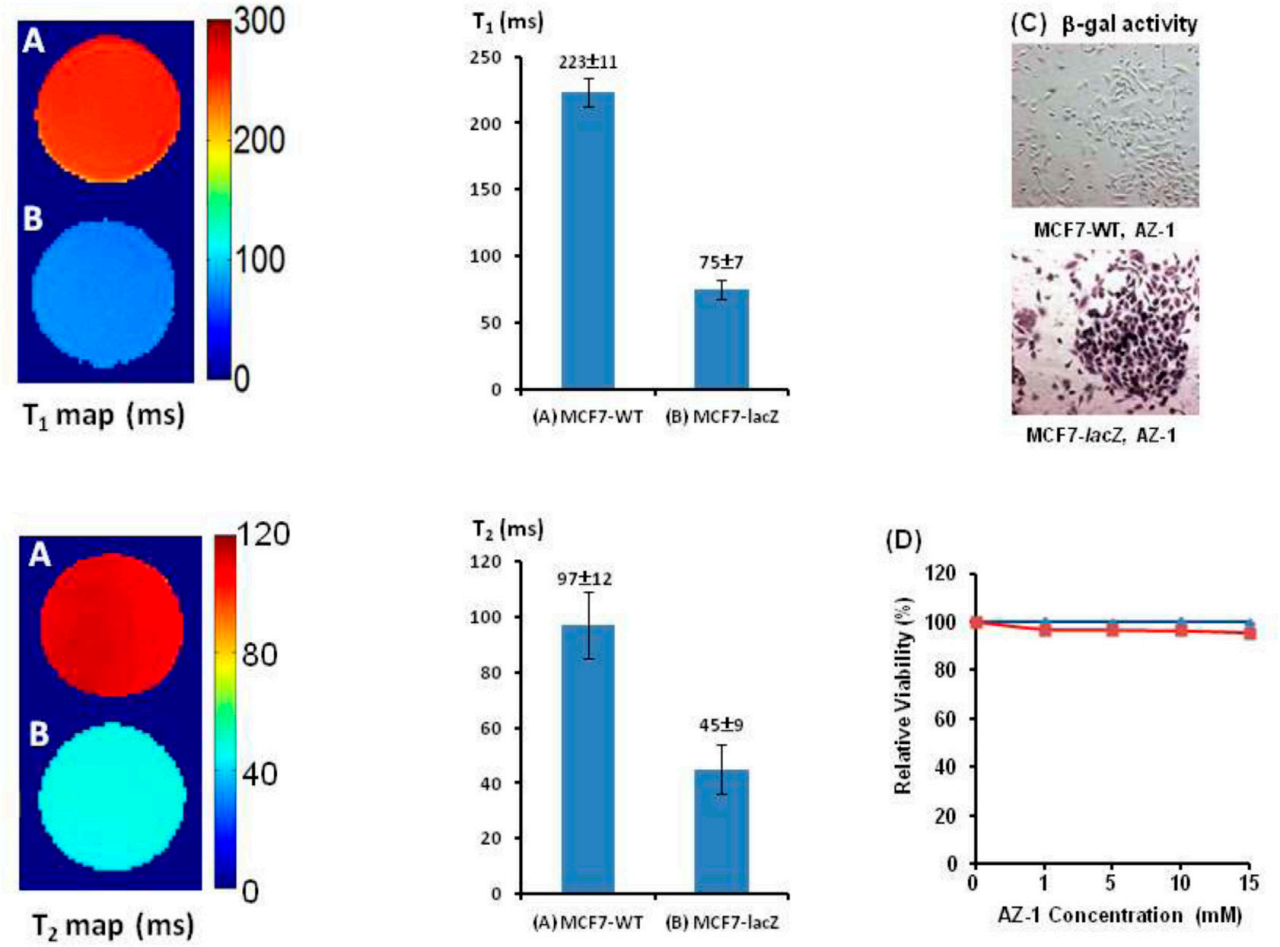
*In vitro* ^1^H-MRI detection of the *lacZ* gene expression in MCF7-*lacZ* cells. ^1^H-MRI acquisition: using the same parameters as in Figure 2. *T*_1_ and *T*_2_ Maps: Solution of alizarin 2-*O*-β-*D*-galactopyranoside **AZ-1** (10.0 mM) and FAC (10.0 mM) in 1:1 (V/V′) DMSO/PBS (0.1 M, pH = 7.4) after incubation 4 h at 37°C under 5% CO_2_/air with 95% humidity with **(A)** MCF7-WT cells [5 × 10^5^, *T*_1(**AZ-1/MCF7-WT/FAC**)_ = 223 ± 11 ms, *T*_2(**AZ-1/MCF7-WT/FAC**)_ = 97 ± 12 ms]; **(B)** MCF7-*lacZ* cells [5 × 10^5^, *T*_1(**AZ-1/MCF7-*lacZ*/FAC**)_ = 75 ± 7 ms, and *T*_2(**AZ-1/MCF7-*lacZ*/FAC**)_ = 45 ± 9 ms]; **(C)** MCF7-WT/MCF7-*lacZ* cells staining by **AZ-1**: dark violet staining confirming intense *lacZ* expression in MCF7-*lacZ* cells with essentially no β-gal activity in MCF7-WT cells. Regional magnification ×100; **(D)** Cytotoxicity: MCF7-WT/MCF7-*lacZ* cells viability *vs.* **AZ-1** in various concentrations in 1:1 (V/V′) DMSO/PBS (0.1 M, pH = 7.4) at 37°C under 5% CO_2_/air with 95% humidity for 72 h, MCF7-WT cells (in blue), and MCF7-*lacZ* cells (in red).

Similarly, after incubation of **AZ-1** (10.0 mM) with MC7-WT cells (5 × 10^5^) and MC7-*lacZ* cells (5 × 10^5^), respectively, in the same conditions as the previous study, the relaxation times were observed to be *T*_1(**AZ-1/MCF7-WT/FAC**)_ = 223 ± 11 ms and *T*_2(**AZ-1/MCF7-WT/FAC**)_ = 97 ± 12 ms in MC7-WT cells, whereas *T*_1(**AZ-1/MCF7-*lacZ*/FAC**)_ = 75 ± 7 ms and *T*_2(**AZ-1/MCF7-*lacZ*/FAC**)_ = 45 ± 9 ms made for MC7-*lacZ* cells (Figures 9A,B), the *T*_1_ and *T*_2_ values are shown as bars adjacent to *T*_1_ and *T*_2_ maps; both illustrated significant differences after the reaction with β-gal at Δ*R*_1_ = 8.85 s^−1^ and Δ*R*_2_ = 11.91 s^−1^. Staining by X-gal, S-gal, and **AZ-1** (dark violet) (Figure 9C) all displayed an intense *lacZ* expression in MC7-*lacZ* cells but with essentially no β-gal activity in MC7-WT cells. Cell viability assays indicated that both **AZ-1** and the released aglycone alizarin have no toxicity to MC7 cells upon the viability above 95% to MC7-WT and MC7-*lacZ* cells at all concentrations tested for 72 h (Figure 9D).

Currently, a Gd-based contrast agent–enhanced ^1^H-MRI has been widely applied for medical diagnosis, offering a noninvasive way to generate anatomical and physiological information while maintaining high spatial and temporal resolution (Terreno et al., 2010; Haris et al., 2015; Wahsner et al., 2019). An Fe-based ^1^H MRI contrast agent, different from the Gd^3+^-based 1H MRI contrast agent with very strong relaxivity, exhibited much shorter relaxation times because of the formation of Fe complexes with the complete coordination of Fe^3+^, eliminating the possibility of inner-sphere to directly coordinate water, leaving outer-sphere and second-sphere coordination water molecules as the only pathways for relaxation (Davies et al., 1996; Richardson et al., 1999; Schwert et al., 2002; Schwert et al., 2005; Haas and Franz, 2009; Kuznik and Wyskocka, 2016). However, an Fe-based contrast agent enhanced ^1^H-MRI has now become a viable alternative because Fe^3+^ is extensively present in the tissues of the human body and is involved in transport, storage, compartmentalization, and excretion mechanisms, while Gd^3+^ is not naturally present in human biochemistry (Beutler, 2004; Weber et al., 2006; Kaplan and Kaplan, 2009; Theil and Goss, 2009). Particularly, cancer cells need a significant amount of Fe^3+^ for rapid replication, so endogenously abundant Fe^3+^ in tumors has been recognized as a molecular target for chemotherapeutic treatments through depleting cancer cellular Fe^3+^ to disrupt cancer cell proliferation and inhibit tumor growth (Fe^3+^-chelation therapy) (Buss et al., 2003; Richardson, 2005). In this study, we introduced exogenous Fe^3+^ with the ultimate goal of developing this approach to hunt the elevated Fe^3+^ level in tumors for the ^1^H-MRI signal generation. Indeed, alizarin has a very high thermodynamic stability constant logβ = 32.21 (Das et al., 1995; Das et al., 2002), indicating its capability of capturing Fe^3+^ from tumor to produce the Fe^3+^–alizarin complex *in situ* while simultaneously generating the ^1^H-MRI signal enhancement (Richardson et al., 1999; Davies et al., 1996; Schwert et al., 2002; Schwert et al., 2005; Haas and Franz, 2009; Kuznik and Wyskocka, 2016). Moreover, alizarin has been known to inhibit human cytomegalovirus replication, HIV-1 RT-associated RDDP, and integrase activities (Esposito et al., 2011). Furthermore, alizarin is the core part of anthraquinones, which constitute numerous antitumor drugs widely applied in the treatment of various neoplasms such as Adriamycin and daunorubicin, and their coordination with Fe^3+^ was shown to diminish cardiotoxicity while improving the antitumor activity in chemotherapy and maintain sound radiosensitizing properties in radiotherapy (Lown, 1993; Nowak and Tarasiuk, 2012; Malik and Müller, 2016). Therefore, this novel molecular platform also indicates the potential for cancer therapy and imaging by utilizing the β-gal responsive turn on pathway to selectively deplete tumor Fe^3+^, resulting in cancer cell cycle arrest and apoptosis while generating ^1^H-MRI contrast enhancement, thereby providing insight into the *lacZ* gene expression, development, location, and magnitude.

## Conclusion

In this study, we present a novel responsive molecular platform for β-gal activity detection using ^1^H-MRI, in which the ^1^H-MRI signal enhancement is specifically generated, localized, and accumulated *in situ* at the β-gal activity site. In conjunction with this design, we have successfully produced and characterized alizarin 2-*O*-β-*d*-galactopyranoside **AZ-1** and alizarin 1,2-di-*O*-β-*d*-galactopyranoside **AZ-2**. We have also demonstrated the feasibility of using **AZ-1** by spontaneous *in situ* formation of paramagnetic Fe^3+^–alizarin complex to assess the β-gal activity in solution with Fe^3+^ ions existence by ^1^H-MRI T1 and T2/T2* relaxation mapping. ^1^H-MRI clearly showed the significant differences in both *T*_1_ and *T*_2_ at WT vs*. lacZ* gene expressing cells in culture after incubation with **AZ-1**, signifying the potential of integrating *T*_1_ and *T*_2_ data together to gain the additional certainty in imaging evaluation and detection reliability of β-gal activity.

## Experimental

### General Methods

NMR spectra were recorded on a Varian Unity INOVA 400 spectrometer (400 MHz for ^1^H, 100 MHz for ^13^C). ^1^H and ^13^C chemical shifts are referenced to TMS as an internal standard with CDCl_3_, or DMSO-*d*_*6*_ as solvents, and chemical shifts are given in ppm. All compounds were characterized by NMR at 25°C. Mass spectra were obtained by positive and negative ESI-MS using a Micromass Q-TOF hybrid quadrupole/time-of-flight instrument (Micromass UK Ltd.). Absorption spectra were taken on a UV-2700 UV-Vis Shimadzu spectrophotometer.

Solutions in organic solvents were dried with anhydrous sodium sulfate and concentrated *in vacuo* below 45°C. 2, 3, 4, 6-Tetra-*O*-acetyl-α-*D*-galactopyranosyl bromide was purchased from the Sigma Chemical Company. β-Gal (E801A) was purchased from the Promega (Madison, WI, United States), and enzymatic reactions were performed at 37°C in the PBS solution (0.1 M, pH = 7.4). Column chromatography was performed on silica gel (200–300 mesh), and silica gel GF_254_ used for analytical TLC was purchased from the Aldrich Chemical Company. The detection was affected by spraying the plates with 5% ethanolic H_2_SO_4_ (followed by heating at 110°C for 10 min) or by direct UV illumination of the plate. The purity of the final products was determined by HPLC with ≥95%.

### Alizarin 2-*O*-2′, 3′, 4′, 6′-Tetra-*O*-Acetyl-β-*D*-Galactopyranoside AZ-M1

A solution of 2, 3, 4, 6-tetra-*O*-acetyl-α-*D*-galactopyranosyl bromide (1.23 g, 3.0 mmol) in CH_2_Cl_2_ (15 ml) was added dropwise to a vigorously stirred CH_2_Cl_2_-H_2_O biphasic mixture (pH 8–9) of alizarin (0.72 g, 3.0 mmol) and tetrabutylammonium bromide (TBAB) (322 mg, 1.0 mmol) in CH_2_Cl_2_-H_2_O (30 ml, 1:1 V/V′) around 1 h at 50°C under N_2_ atmosphere, with stirring continued for an additional 4–5 h until TLC showed that the reaction was completed. The product was extracted with CH_2_Cl_2_ (4 × 30 ml) and subsequently washed (H_2_O), dried (Na_2_SO_4_), and evaporated under reduced pressure to give a syrup, which was purified by column chromatography on silica gel to give alizarin 2-*O*-2′, 3′, 4′, 6′-tetra-*O*-acetyl-β-*D*-galactopyranoside **AZ-M1**.

### Alizarin 1,2-Di-*O*-2′, 3′, 4′, 6′-Tetra-*O*-Acetyl-β-*D*-Galactopyranoside AZ-M2

A solution of 2, 3, 4, 6-tetra-*O*-acetyl-α-*D*-galactopyranosyl bromide (2.71 g, 6.6 mmol) in CH_2_Cl_2_ (30 ml) was added dropwise to a vigorously stirred CH_2_Cl_2_-H_2_O biphasic mixture (pH 10–11) of alizarin (0.72 g, 3.0 mmol) and TBAB (322 mg, 1.0 mmol) in CH_2_Cl_2_-H_2_O (30 ml, 1:1 V/V′) around 1 h at 55°C under N_2_ atmosphere, with stirring continued for an additional 4–5 h until TLC showed that the reaction was completed. The product was extracted with CH_2_Cl_2_ (4 × 40 ml) and subsequently washed (H_2_O), dried (Na_2_SO_4_), and evaporated under reduced pressure to give a syrup, which was purified by column chromatography on silica gel to give alizarin 1,2-di-*O*-2′, 3′, 4′, 6′-tetra-*O*-acetyl-β-*D*-galactopyranoside **AZ-M2**.

### Alizarin 2-*O*-β-*D*-Galactopyranoside AZ-1 and Alizarin 1,2-Di-*O*-β-*D*-Galactopyranoside AZ-2

General procedure: A solution of alizarin 2-*O*-2′, 3′, 4′, 6′-tetra-*O*-acetyl-β-*D*-galactopyranoside **AZ-M1** or alizarin 1,2-di-*O*-2′, 3′, 4′, 6′-tetra-*O*-acetyl-β-*D*-galactopyranoside **AZ-M2** (1.30 g) in anhydrous MeOH (120 ml) containing 0.5 M NH_3_ was vigorously stirred from 0°C to room temperature overnight until TLC showed that the reaction was complete and then evaporated to dryness *in vacuo*. Chromatography of the crude syrup on silica gel with ethyl acetate-methanol afforded the corresponding alizarin 2-*O*-β-*D*-galactopyranoside **AZ-1** and alizarin 1,2-di-*O*-β-*D*-galactopyranoside **AZ-2** in high yields.

### MRI

MRI studies were performed using a 4.7T horizontal bore magnet or a 9.4T vertical bore magnet equipped with a Varian INOVA Unity system (Palo Alto, CA, United States). *T*_1_ and *T*_2_ (or *T*_2_^*^) maps were acquired using a spin echo (or gradient echo) sequence with varying repetition times (TRs) or echo times (TEs), respectively. The raw data were acquired using a centric *k*-space reordering scheme, followed by the phase encoding steps with higher phase encoding gradient amplitudes. Data acquisition parameters of the FLASH readout were TR/TE/Flip angle = 10 ms/5 ms/10°. The standard multi-echo Carr–Purcell–Meiboom–Gill pulse sequence was used for measuring *T*_2_ from a single echo train. The *T*_2_ and *T*_2_^*^ maps were obtained on a voxel-by-voxel basis using a nonlinear least-squares fit equation M = M_0_e^−TE/T2^ from the images taken at each echo time. Images were reconstructed and analyzed by using MatLab (MathWorks, Natick, MA).

### *lacZ* Transfection in Human Tumor Cells

The *E. coli lacZ* gene (from pSV-β-gal vector, Promega, Madison, WI, United States) was inserted into a high expression human cytomegalovirus (CMV) immediate early enhancer/promoter vector phCMV (Gene Therapy Systems, San Diego, CA, United States), producing a recombinant vector phCMV/*lacZ*. This was used to transfect wild-type MCF7 (human breast cancer) and PC3 (human prostate cancer) cells (ATCC, Manassas, VA, United States) using GenePORTER2 (Gene Therapy Systems, Genlantis, Inc., San Diego, CA, United States). The highest β-gal expressing colony was selected using the antibiotic G418 disulfate (800 μg/ml, Research Products International Corp, Mt Prospect, IL, United States), and G418 (200 μg/ml) was also included for routine culture. The cells were maintained in Dulbecco’s modified Eagle’s medium (DMEM, Mediatech Inc., Herndon, VA, United States) containing 10% fetal bovine serum (FBS, 0.1 M, pH = 7.4, Atlanta Biologicals, Inc., Lawrenceville, GA, United States) with 100 units/mL of penicillin and 100 units/mL streptomycin, and cultured in a humidified 5% CO_2_ incubator at 37°C. The β-gal activity of *lacZ*-transfected tumor cells was measured using a β-Gal Assay Kit with *o*-nitrophenyl-β-*D*-galactopyranoside (Promega, Madison, WI, United States) and confirmed by X-gal or S-gal staining. Cells were fixed in PBS plus 0.5% glutaraldehyde (5 min) and rinsed in PBS prior to staining. Staining was performed using standard procedures for 2 h at 37°C in PBS plus 1 mg/ml X-gal (Sigma, St. Louis, MO, United States), 1 mM MgCl_2_, 5 mM K_3_Fe(CN)_6_, and 5 mM K_4_Fe(CN)_6_ or with 1.5 mg/mL S-gal (Sigma, St. Louis, MO, United States) and 2.5 mg/ml FAC.

### Western Blot

The protein extracted from the wild-type and *lacZ*-expressing MCF7 and PC3 cancer cells was quantified using the Bradford method by a protein assay (Bio-Rad, Hercules, CA, United States). Protein (30 μg) was added to each well, separated by 10% sodium dodecyl sulfate-polyacrylamide gel electrophoresis, and transferred to a polyvinylidene fluoride membrane. A primary monoclonal anti–β-gal antibody (Promega, Madison, WI, United States) and anti-actin antibody (Sigma, St. Louis, MO, United States) were used as probes at a dilution of 1:5,000, with the reacting protein detected using a horseradish peroxidase–conjugated secondary antibody and ECL detection (Amersham, Piscataway, NJ, United States).

### Cytotoxicity

The cytotoxicity for the free β-*D*-galactopyranoside **AZ-1** and the released aglycone alizarine was assessed in both wild-type and *lacZ-* expressing MCF7 and PC3 cells using a colorimetric CellTiter 96 Aq_ueous_ Non-Radioactive Cell Proliferation Assay (MTS) (Promega, Madison, WI, United States). Assays were performed in triplicate using 24-well plates seeded with 10^3^ cells per well in 500 μL of RPMI-1640 without phenol red and supplemented with 10% FCS and 2 mM glutamine (Urano et al., 2005; Kamiya et al., 2007).

## Data Availability

The datasets presented in this study can be found in online repositories. The names of the repository/repositories and accession number(s) can be found in the article/Supplementary Material.

## Abbreviations

NMR, nuclear magnetic resonance; MRS, magnetic resonance spectroscopy; MRI, magnetic resonance imaging; TR, repetition time; TE, echo time; β-gal, β-galactosidase; FAC, ferric ammonium citrate, TBAB, tetrabutylammonium bromide; CH2Cl2, dichloromethane; HRMS, high-resolution mass spectrometry; PBS, phosphate-buffered saline; X-Gal, 5-bromo-4-chloro-3-indolyl-β-D-galactopyranoside; S-Gal, 3,4-cyclohexenoesculetin β-D-galactopyranoside; DMSO, dimethyl sulfoxide; TLC, thin-layer chromatography.
