# Supplementary material for: In Situ Generated Novel 1H MRI Reporter for β-Galactosidase Activity Detection and Visualization in Living Tumor Cells
Source: Front Chem. 2021 Jul 15;9:709581. doi: 10.3389/fchem.2021.709581 (PMC8321238; doi:10.3389/fchem.2021.709581)
Supplement: Supplementary file 1 [file DataSheet1.PDF]

Supporting Information

For

In Situ Generated Novel ^{1}H MRI Reporters for β-Galactosidase Activity Detection and Visualization in Living Tumor Cells

Shuo Gao,[1] Lei Zhao,[1†] Zhiqiang Fan,[1†] Vikram D. Kodibagkar,[2] Li Liu,[3] Hanqin Wang,[1] Hong Xu,[1] Mingli Tu,[1] Bifu Hu,[1] Chuanbin Cao,[1] Zhenjian Zhang,[1] Jian-Xin Yu,[1,4]*

[1] Center of Translational Medicine, 5th School of Medicine/Suizhou Central Hospital, Hubei University of Medicine, 8 Wenhua Park Road, Suizhou, Hubei 441300, China

[2] School of Biological and Health Systems Engineering, Arizona State University, Tempe, AZ 85287-9709, USA

[3] Department of Radiology, University of Texas Southwestern Medical Center at Dallas, 5323 Harry Hines Blvd., Dallas, Texas 75390-9058, USA

[4] Biomedical Research Institute, Hubei University of Medicine, 30 South Renmin Road, Shiyan, Hubei 442000, China

† Equal Contributions.

CONTENTS:

1. Supporting figures

(1) **Figure S1**, The numbering of structures **AZ-M1** and **AZ-1**.

(2) **Figure S2**, The numbering of structures **AZ-M2** and **AZ-2**.

(3) **Figure S3**, The formation of molecular tweezer complex **AZ-2/Fe^{3+}**.

2. Fe^{3+}-alizarin complex preparation.

3. Molecular characterization of AZ-M1, AZ-M2, AZ-1 and AZ-2.

4. Figures S1-S13, NMR spectra of AZ-M1, AZ-M2, AZ-1 and AZ-2.

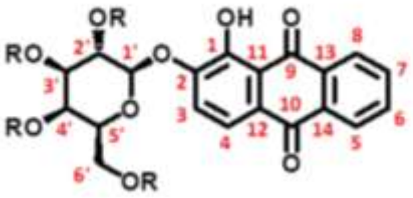

AZ-M1 R = Ac
AZ-1 R = H

Figure S1. The numbering of structures **AZ-M1** and **AZ-1**.

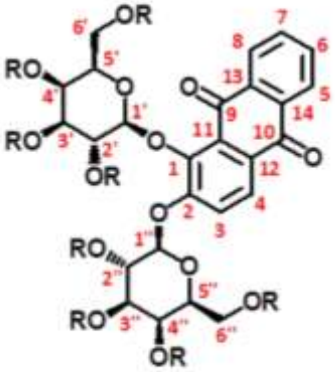

AZ-M2 R = Ac
AZ-2 R = H

Figure S2. The numbering of structures **AZ-M2** and **AZ-2**.

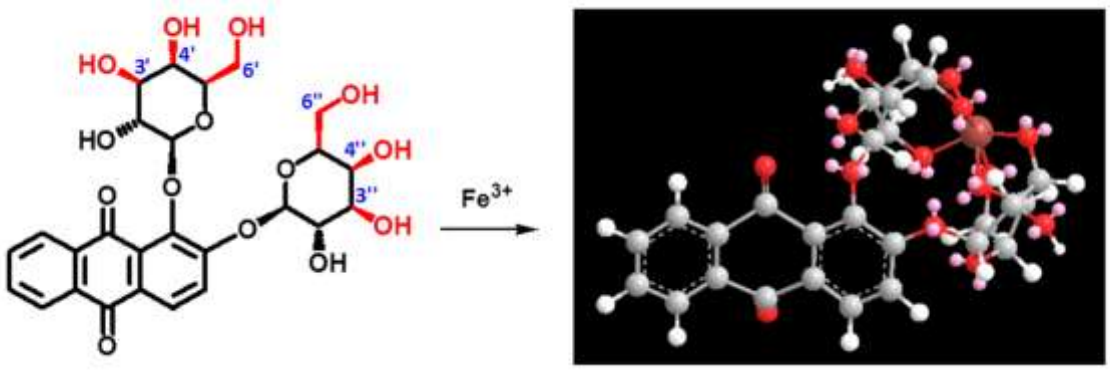

AZ-2 AZ-2/Fe^{3+} Complex

Figure S3. The formation of molecular tweezer complex **AZ-2/Fe^{3+}** (Modeling was calculated from the computed minimized energy conformation using Chem3D 8.0, MM2 as the force field).

Fe^{3+}-alizarin complex preparation. To a solution of alizarin (721 mg, 3.0 mmol) and Et_3N (323 mg, 3.2 mmol) in anhydrous MeOH (80 mL) was dropped a solution of $Fe(ClO_4)_3{\cdot}6H_2O$ (462 mg, 1.0 mmol) in anhydrous MeOH (50 mL) with stirring at gently refluxing under N_2 for 30 min. Upon cooling, a fine brown precipitate was obtained, which was filtered off, washed with EtOH then Et_2O, and dried in the vacuum.

Fe^{3+}-Alizarin Complex, $[Fe(Alizarin\text{-}H)_3]{\cdot}2H_2O$ (765 mg) as brown powders. Anal. Calcd. for $C_{42}H_{25}FeO_{14}$ (%): C, 62.32, H, 3.11; Found: C, 62.27, H, 3.06.

Alizarin 2-*O*-2', 3', 4', 6'-Tetra-*O*-Acetyl-β-*D*-Galactopyranoside AZ-M1 (1.34 g, 78%) as brown syrup, R_f 0.62 (3**:**2 cyclohexane-EtOAc). δ_H ($CDCl_3$): 12.81 (1H, s, 1-OH, exchangeable with D_2O), 7.48 (1H, d, $J_{3,4}$ = 8.34 Hz, Ar-H_3), 7.78 (1H, d, Ar-H_4), 8.29 (1H, dd, $J_{5,6}$ = 6.84 Hz, $J_{5,7}$ = 1.20 Hz, Ar-H_5), 7.81 (1H, dd, $J_{6,7}$ = 1.56 Hz, Ar-H_6), 7.82 (1H, dd, $J_{7,8}$ = 6.96 Hz, Ar-H_7), 8.28 (1H, dd, $J_{6,8}$ = 2.04 Hz, Ar-H_8), 5.15 (1H, d, $J_{1',2'}$ = 8.10 Hz, H-1'), 5.60 (1H, dd, $J_{2',3'}$ = 10.44 Hz, H-2'), 5.17 (1H, dd, $J_{3',4'}$ = 3.42 Hz, H-3'), 5.49 (1H, d, $J_{4',5'}$ = 3.06 Hz, H-4'), 4.10 (1H, t, $J_{5',6a'}$ = $J_{5',6b'}$ = 6.60 Hz, H-5'), 4.25 (1H, dd, $J_{6a',6b'}$ = 11.37 Hz, H-6a'), 4.20 (1H, dd, H-6b'), 2.21, 2.15, 2.07, 2.04 (12 H, 4 s, 4 × CH_3CO) ppm. δ_C ($CDCl_3$): 150.38 (C-1), 153.85 (C-2), 119.98 (C-3), 124.50 (C-4), 127.46 (C-5), 134.92 (C-6), 133.70 (C-7), 133.17 (C-8), 181.48 (C-9), 188.90 (C-10), 117.06 (C-11), 126.97 (C-12), 128.44 (C-13), 134.07 (C-14), 100.46 (C-1'), 68.36 (C-2'), 70.53 (C-3'), 66.84 (C-4'), 71.40 (C-5'), 61.37 (C-6'), 170.33, 170.21, 170.12, 169.62 (4 × CH_3**C**O), 21.81, 20.67, 20.66, 20.60 (4 × **C**H_3CO) ppm.

HRMS: $[M+H]^+$, $C_{28}H_{27}O_{13}$, Calcd: 571.5062, Found: 571.5037; $[M+Na]^+$, $C_{28}H_{26}O_{13}Na$, Calcd: 593.4880, Found: 593.4853.

Alizarin 1,2-Di-*O*-2', 3', 4', 6'-Tetra-*O*-Acetyl-β-*D*-Galactopyranoside AZ-M2 (1.68 g, 62%) as brown syrup, R_f 0.51 (1:2 cyclohexane-EtOAc). δ_H ($CDCl_3$): 7.47 (1H, m, Ar-H_3), 7.71 (1H, m, Ar-H_4), 8.16 (2H, m, Ar-$H_{5,8}$), 8.06 (2H, m, Ar-$H_{6,7}$), 5.24 (1H, d, $J_{1',2'}$ = 7.58 Hz, H-1'), 5.26 (1H, d, $J_{1'',2''}$ = 7.65 Hz, H-1''), 5.49 (1H, dd, $J_{2',3'}$ = 9.72 Hz, H-2'), 5.59 (1H, dd, $J_{2'',3''}$ = 10.08 Hz, H-2''), 5.08 (1H, m, H-3'), 5.12 (1H, m, H-3''), 5.34 (1H, m, H-4'), 5.43 (1H, m, H-4''), 3.92 (1H, m, H-5'), 4.00 (1H, m, H-5''), 4.03 (2H, m, H-6'), 4.16 (2H, m, H-6''), 2.17 - 1.96 (24 H, m, 8 × CH_3CO) ppm. δ_C ($CDCl_3$): 145.02 (C-1), 155.20 (C-2), 122.64 (C-3), 124.97 (C-4), 127.30 (C-5), 133.64 (C-6), 132.38 (C-7), 127.60 (C-8), 181.51 (C-9), 182.13 (C-10), 126.62 (C-11), 130.17 (C-12), 134.18 (C-13), 134.91 (C-14), 99.66 (C-1'), 100.44 (C-1''), 68.66 (C-2'), 69.95 (C-2''), 70.47 (C-3'), 71.15 (C-3''), 66.75 (C-4'), 67.00 (C-4''), 71.35 (C-5'), 71.50 (C-5''), 60.99 (C-6') 61.40 (C-6''), 171.05, 170.43, 170.18, 170.14, 170.11, 169.97, 169.57, 169.36 (8 × $CH_3\mathbf{C}O$), 20.99, 20.86, 20.78, 20.64, 20.60, 20.59, 20.54, 20.43 (8 × $\mathbf{C}H_3CO$) ppm.

HRMS: $[M]^+$, $C_{42}H_{44}O_{22}$, Calcd: 900.7856, Found: 900.7837; $[M+Na]^+$, $C_{42}H_{44}O_{22}Na$, Calcd: 923.7753, Found: 923.7732.

Alizarin 2-*O*-β-*D*-Galactopyranoside AZ-1 (872 mg, 91%) as brown foam solid, R_f 0.48 (1:4 MeOH-EtOAc). δ_H (DMSO-d_6): 15.26 (1H, s, 1-OH, exchangeable with D_2O), 8.45 - 6.73 (6H, m, Ar-$H_{3,4,5,6,7,8}$), 4.97 (1H, d, $J_{1',2'}$ = 7.74 Hz, H-1'), 3.77 - 3.50 (6H, m, H-2',3',4',5',6'), 5.10 - 4.30 (4H, br, HO-2', 3', 4', 6', exchangeable with D_2O) ppm. δ_C (DMSO-d_6): 161.02 (C-1), 167.69 (C-2), 116.65 (C-3), 117.26 (C-4), 125.28 (C-5), 155.42 (C-6), 133.36 (C-7), 132.67 (C-8), 171.75 (C-9), 180.26 (C-10), 110.65 (C-11), 125.04 (C-12), 127.25 (C-13), 133.69 (C-14), 100.79 (C-1'), 70.32 (C-2'), 73.49 (C-3'), 68.23 (C-4'), 75.82 (C-5'), 60.51 (C-6') ppm.

HRMS: $[M+H]^+$, $C_{20}H_{19}O_9$, Calcd: 403.3595, Found: 403.3559; $[M+Na]^+$, $C_{20}H_{18}O_9Na$, Calcd: 425.3413, Found: 425.3380.

Alizarin 1,2-Di-*O*-β-*D*-Galactopyranoside AZ-2 (765 mg, 94%) as brown powder, R_f 0.42 (1**:**2 MeOH-EtOAc). δ_H (DMSO-d_6): 7.65 (1H, d, $J_{3,4}$ = 8.76 Hz, Ar-H_3), 7.97 (1H, d, Ar-H_4), 8.10 (1H, d, $J_{5,6}$ = 7.38 Hz, Ar-H_5), 7.87 (1H, t, $J_{6,7}$ = 7.38 Hz, Ar-H_6), 7.83 (1H, t, $J_{7,8}$ = 7.32 Hz, Ar-H_7), 8.08 (1H, d, Ar-H_8), 5.02 (1H, d, $J_{1',2'}$ = 7.62 Hz, H-1'), 5.08 (1H, d, $J_{1'',2''}$ = 7.62 Hz, H-1''), 3.72 (1H, dd, $J_{2',3'}$ = 8.16 Hz, H-2'), 3.79 (1H, dd, $J_{2'',3''}$ = 8.52 Hz, H-2''), 3.38 (1H, dd, $J_{3',4'}$ = 5.94 Hz, H-3'), 3.60 (1H, dd, $J_{3'',4''}$ = 5.52 Hz, H-3''), 3.69 (1H, m, H-4'), 3.77 (1H, m, H-4''), 3.29 (2H, m, H-5', H-5''), 3.49 (4H, m, H-6', H-6''), 4.92 - 4.40 (8H, br, HO-2', 2'', 3', 3'', 4', 4'', 6', 6'', exchangeable with D_2O) ppm. δ_C (DMSO-d_6): 145.14 (C-1), 156.03 (C-2), 120.47 (C-3), 124.27 (C-4), 126.73 (C-5), 132.25 (C-6), 133.80 (C-7), 127.70 (C-8), 181.69 (C-9), 181.85 (C-10), 126.13 (C-11), 127.76 (C-12), 134.36 (C-13), 135.08 (C-14), 101.50 (C-1'),103.80 (C-1''), 70.53 (C-2'), 71.74 (C-2''), 72.89 (C-3'), 73.27 (C-3''), 67.96 (C-4'), 68.18 (C-4''), 75.64 (C-5'), 75.97 (C-5''), 60.07 (C-6'), 60.47 (C-6'') ppm.

HRMS: $[M+H]^+$, $C_{26}H_{29}O_{14}$, Calcd: 565.5001, Found: 565.4968; $[M+Na]^+$, $C_{26}H_{28}O_{14}Na$, Calcd: 587.4819, Found: 587.4791.

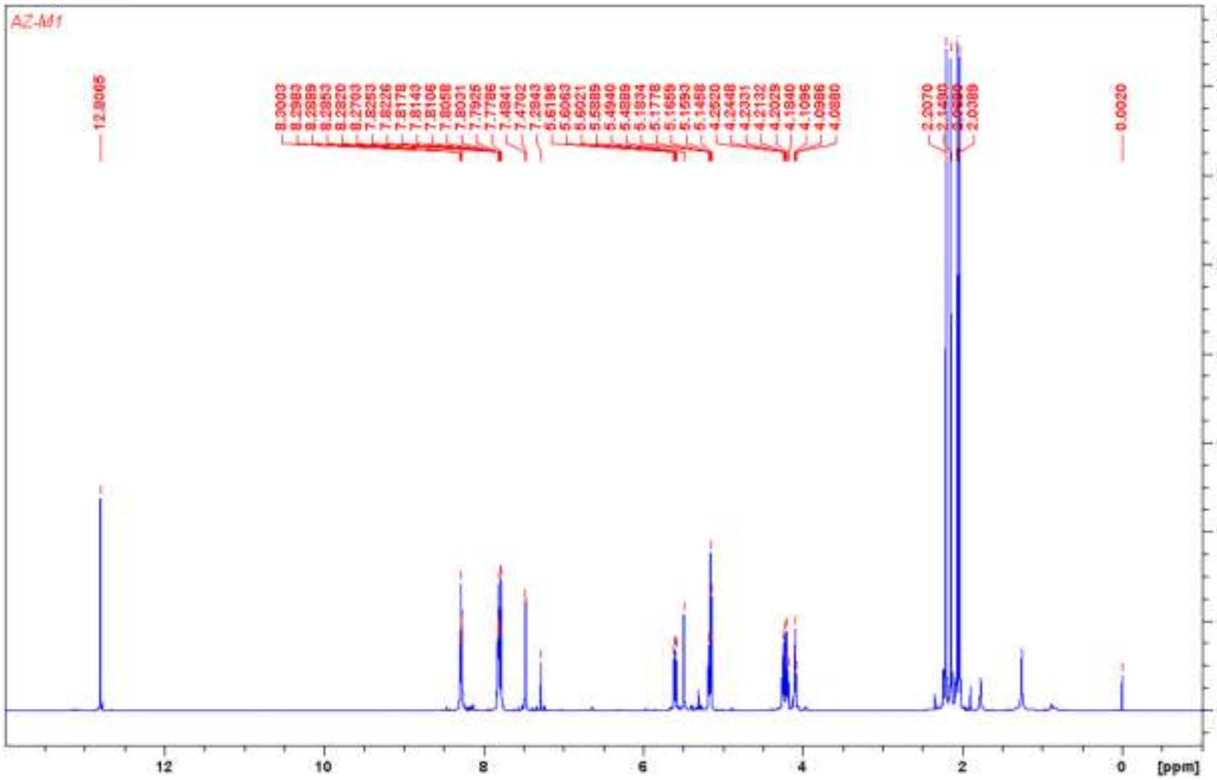

Figure S4. ^{1}H NMR spectrum of **AZ-M1**.

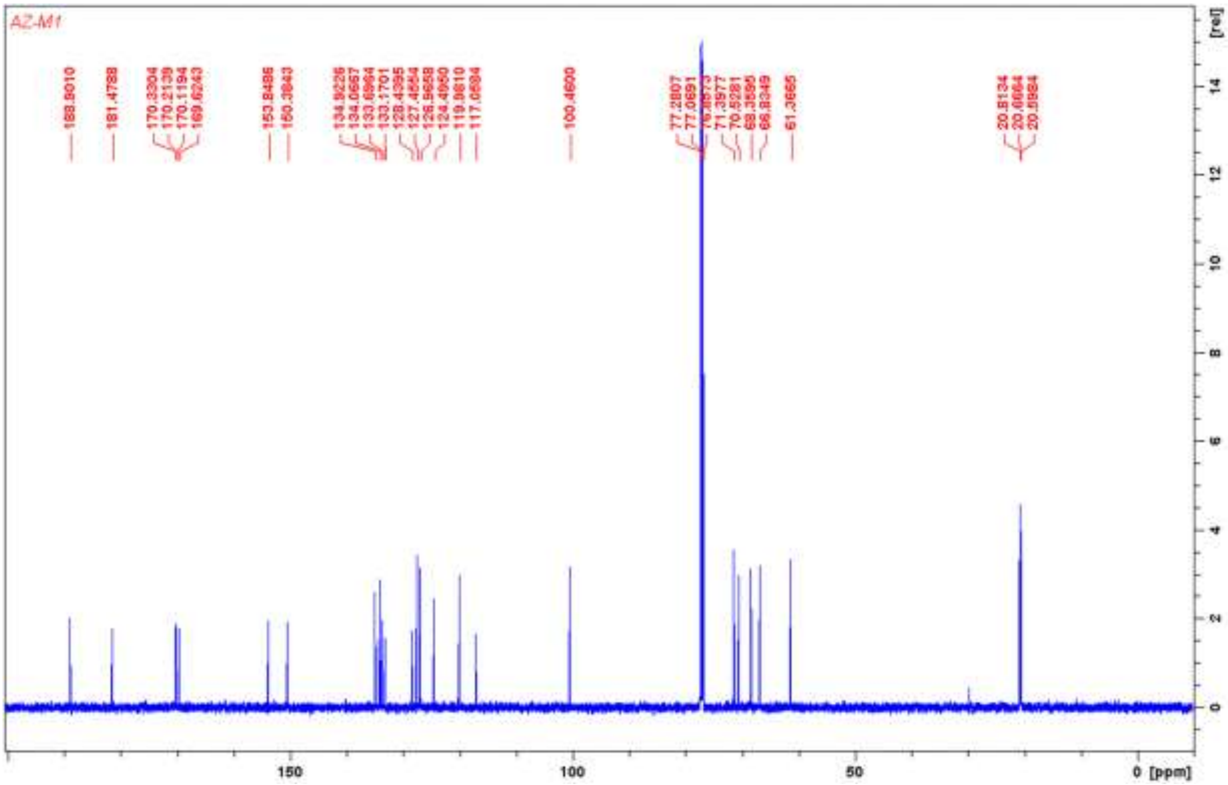

Figure S5. ^{13}C NMR spectrum of **AZ-M1**.

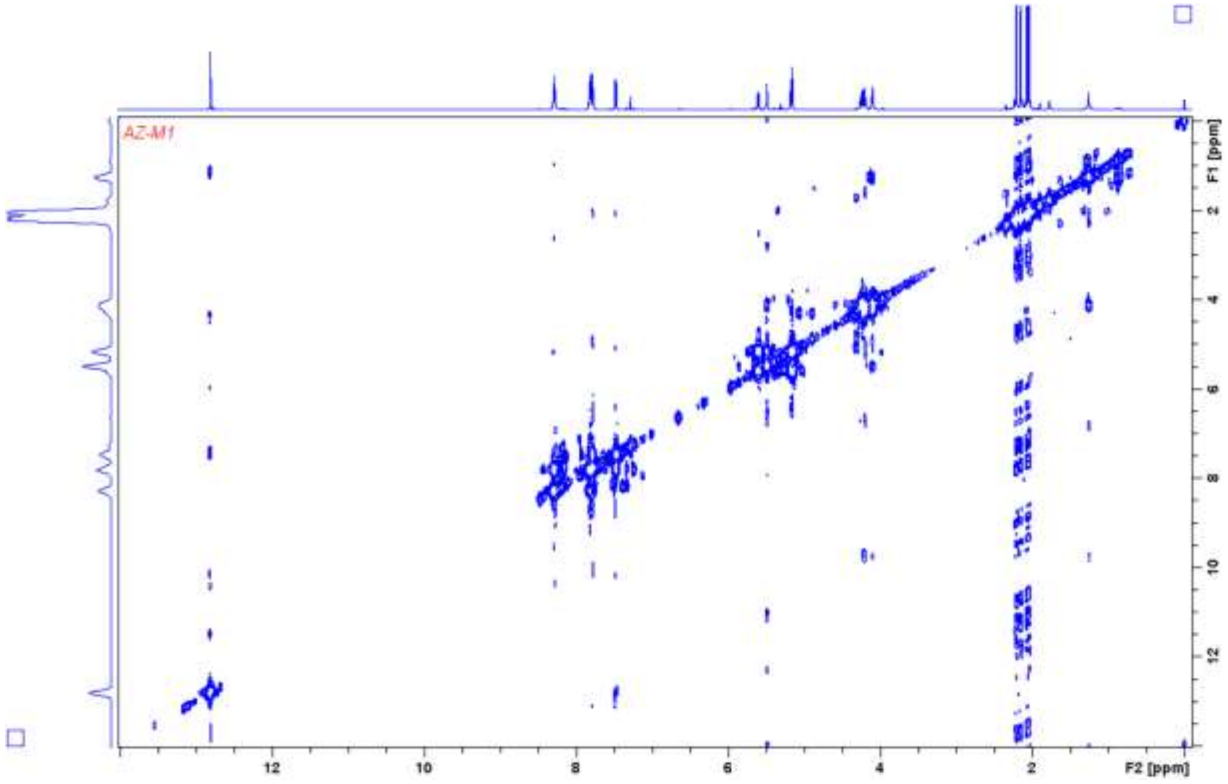

Figure S6. COSY spectrum of **AZ-M1**.

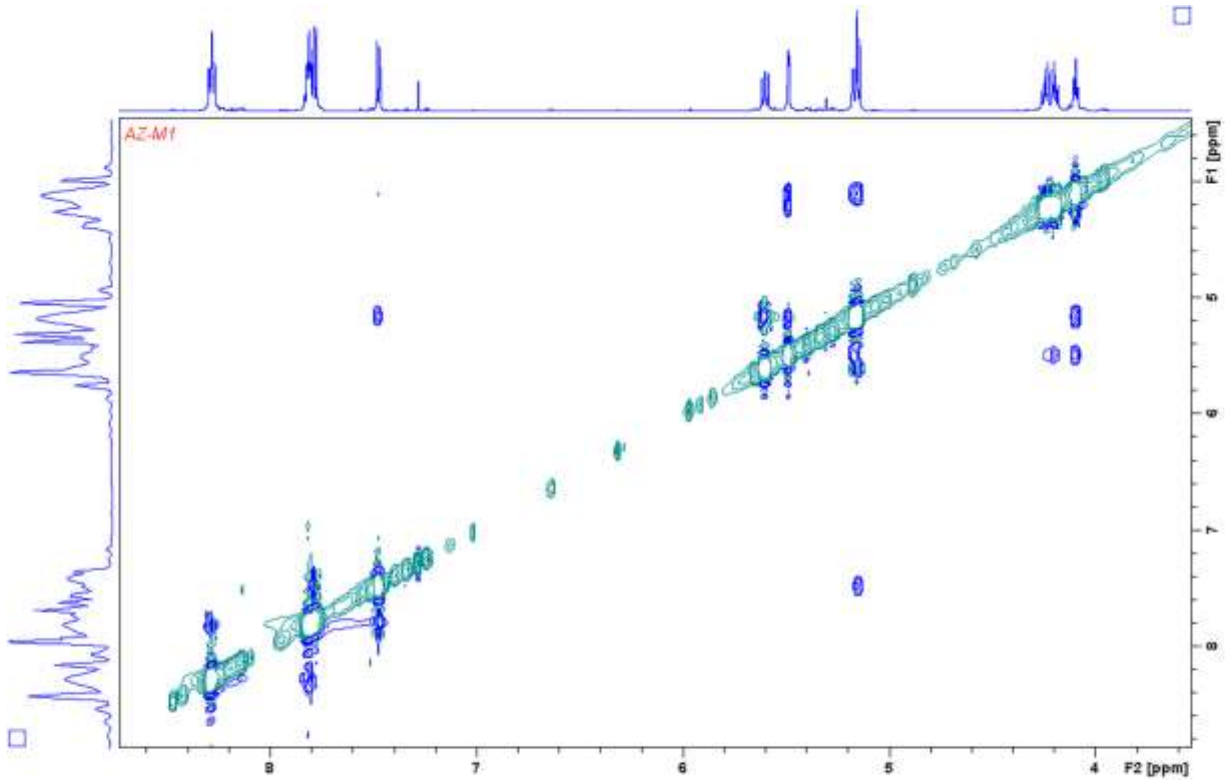

Figure S7. NOESY spectrum of **AZ-M1**.

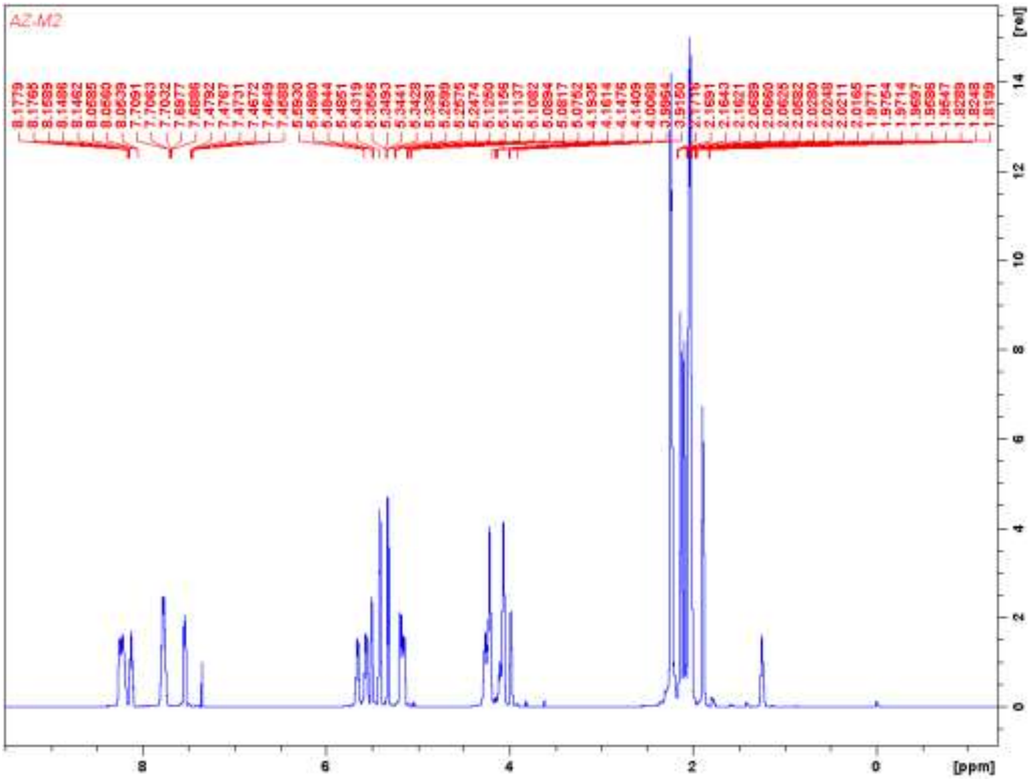

Figure S8. ^{1}H NMR spectrum of **AZ-M2**.

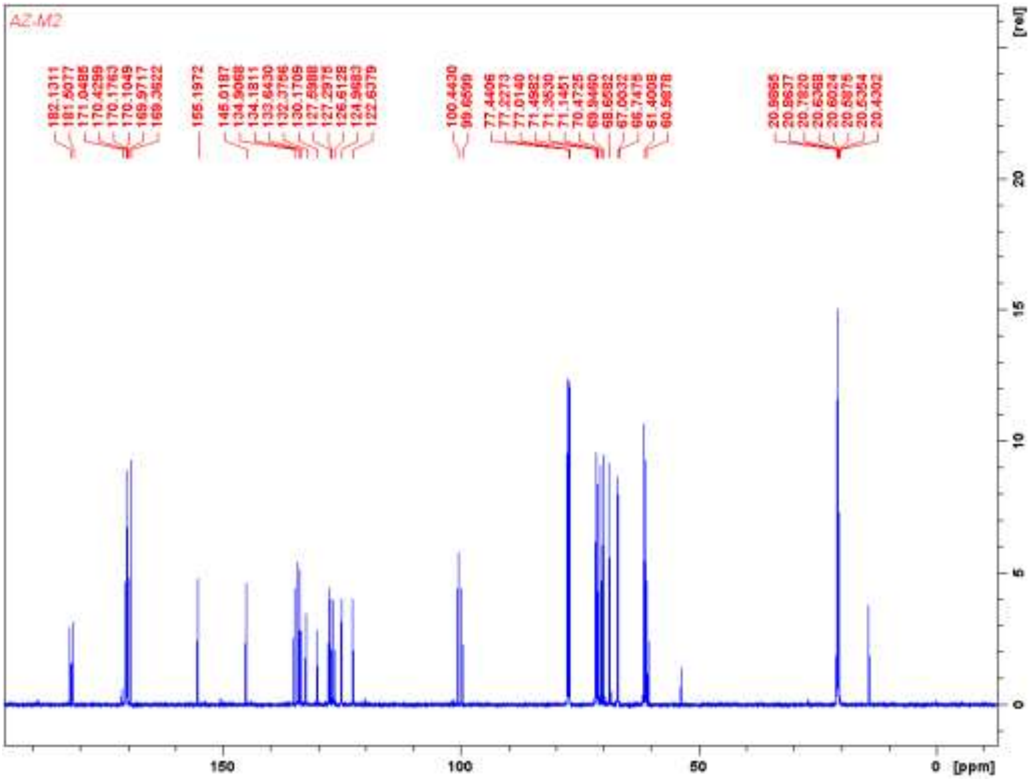

Figure S9. ^{13}C NMR spectrum of **AZ-M2**.

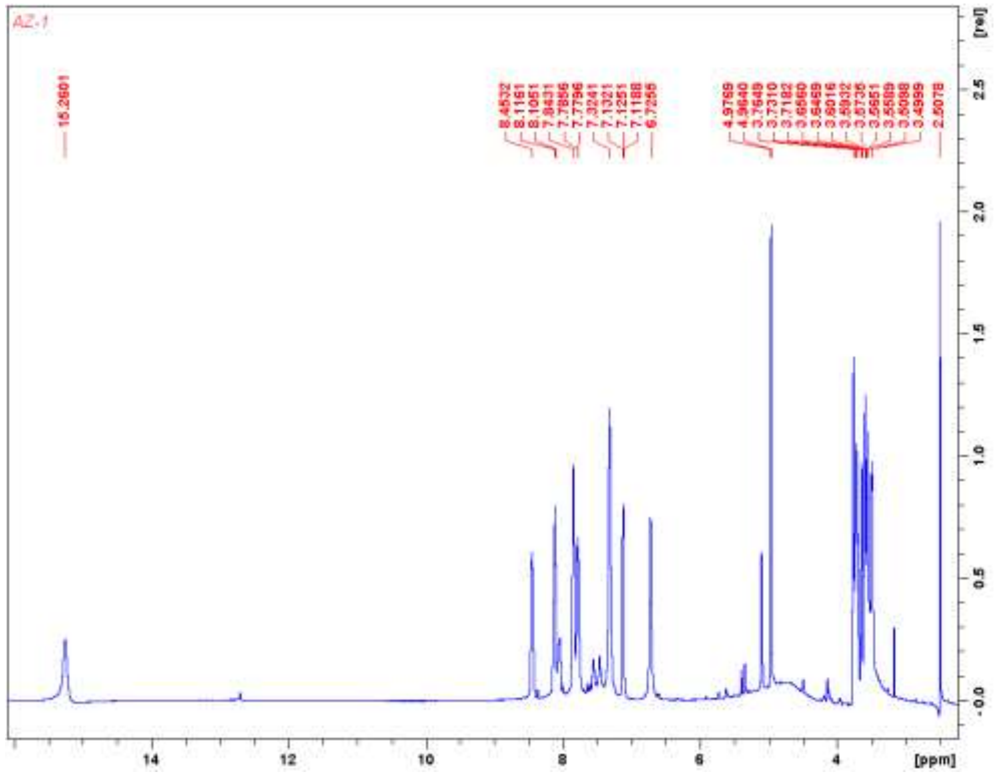

Figure S10. ^{1}H NMR spectrum of **AZ-1**.

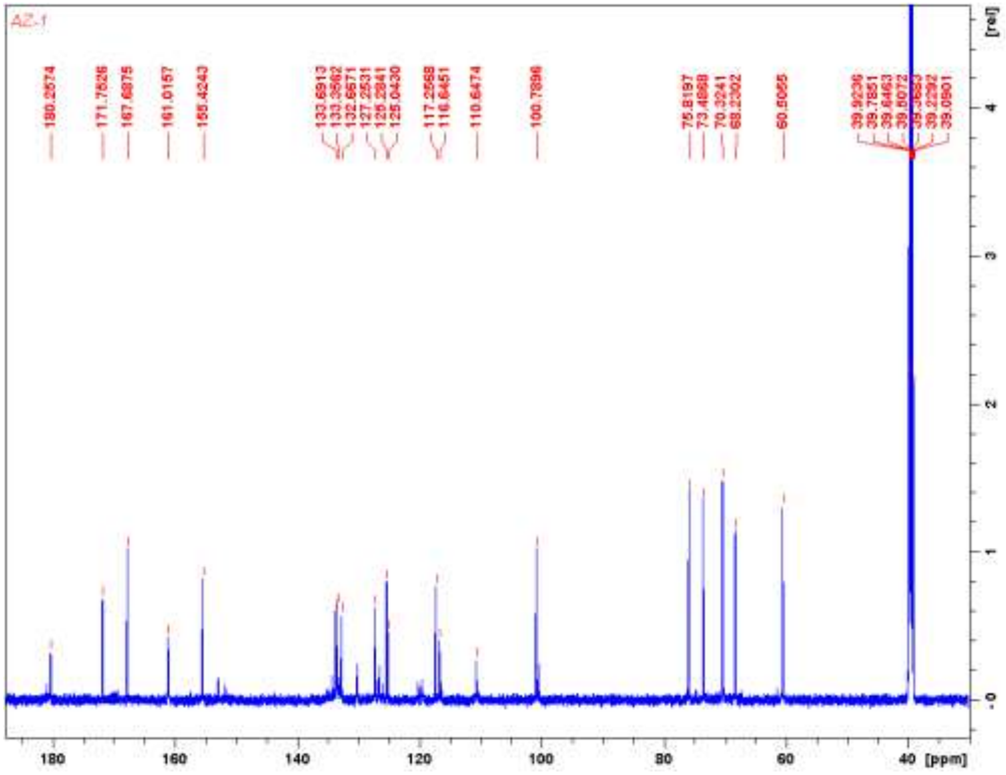

Figure S11. ^{13}C NMR spectrum of **AZ-1**.

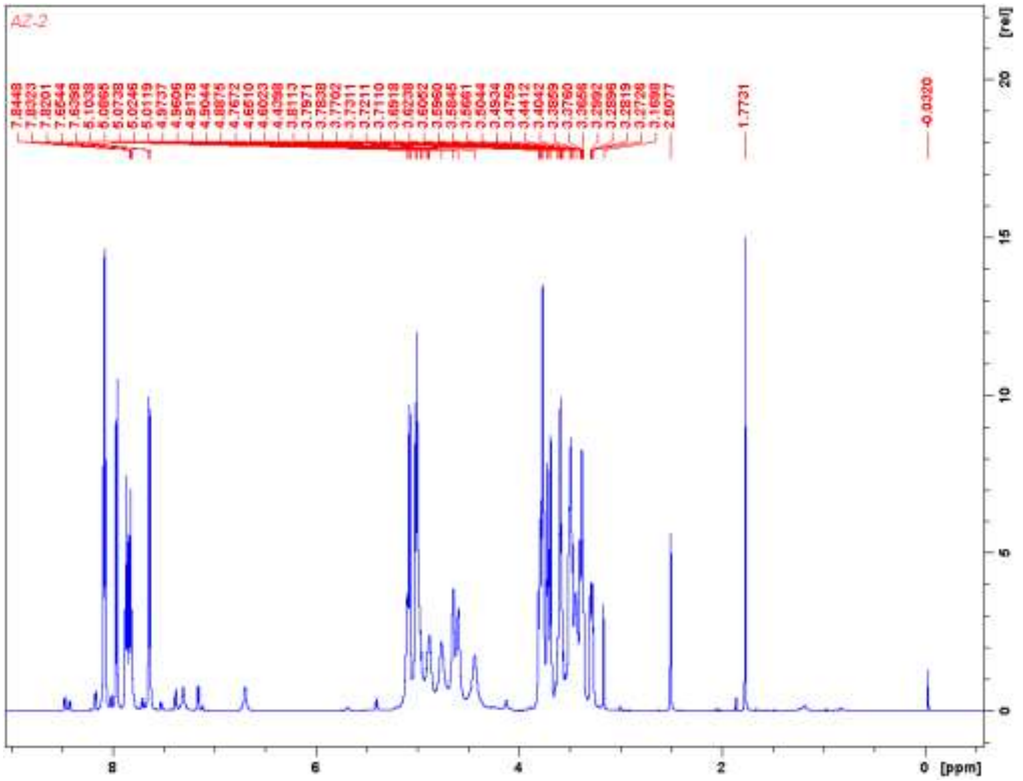

Figure S12. ^{1}H NMR spectrum of **AZ-2**.

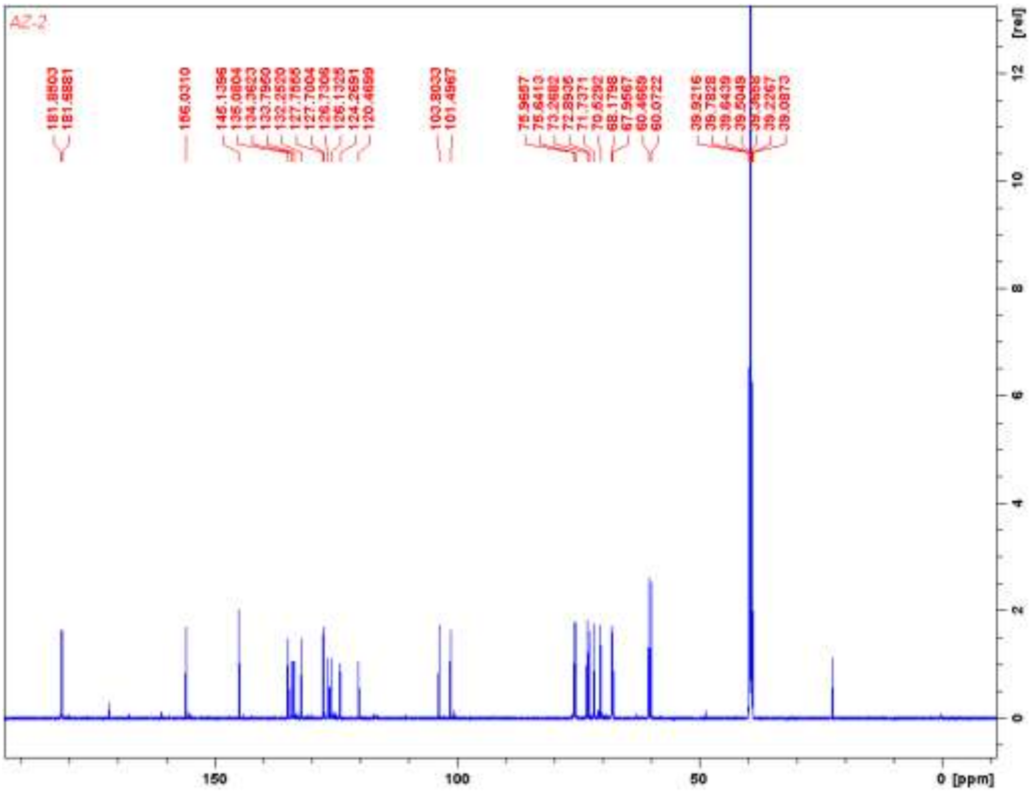

Figure S13. ^{13}C NMR spectrum of **AZ-2**.
