# Supplementary material for: In Situ Generated Novel 1H MRI Reporter for β-Galactosidase Activity Detection and Visualization in Living Tumor Cells
Source: Front Chem. 2021 Jul 15;9:709581. doi: 10.3389/fchem.2021.709581 (PMC8321238; doi:10.3389/fchem.2021.709581)
Supplement: Supplementary file 2 [file DataSheet2.zip › Raw image data for manuscript #709581.pptx]

## Slide 1
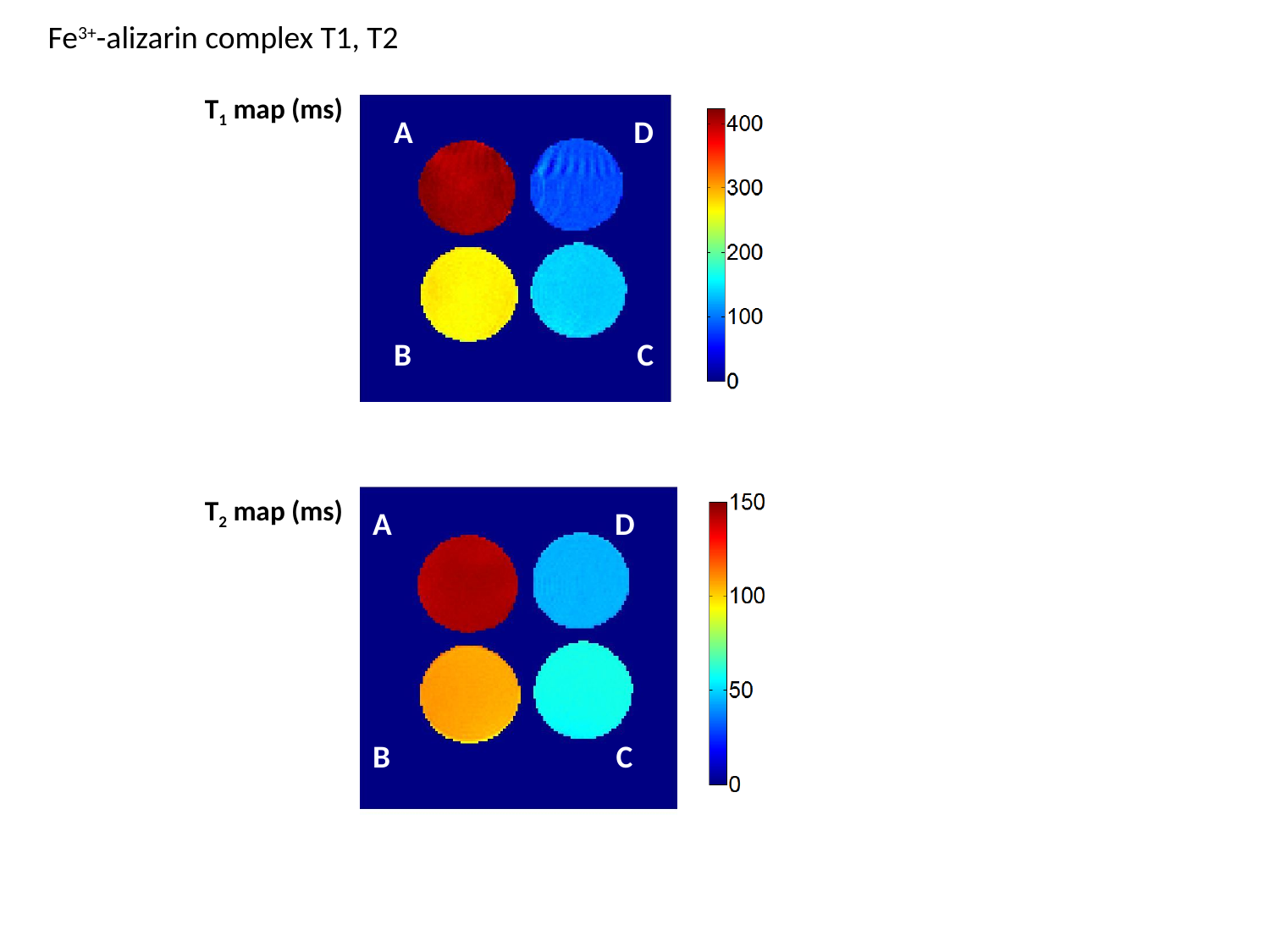

Fe3+-alizarin complex T1, T2
T1 map (ms)
A
D
B
C
T2 map (ms)
A
D
B
C

## Slide 2
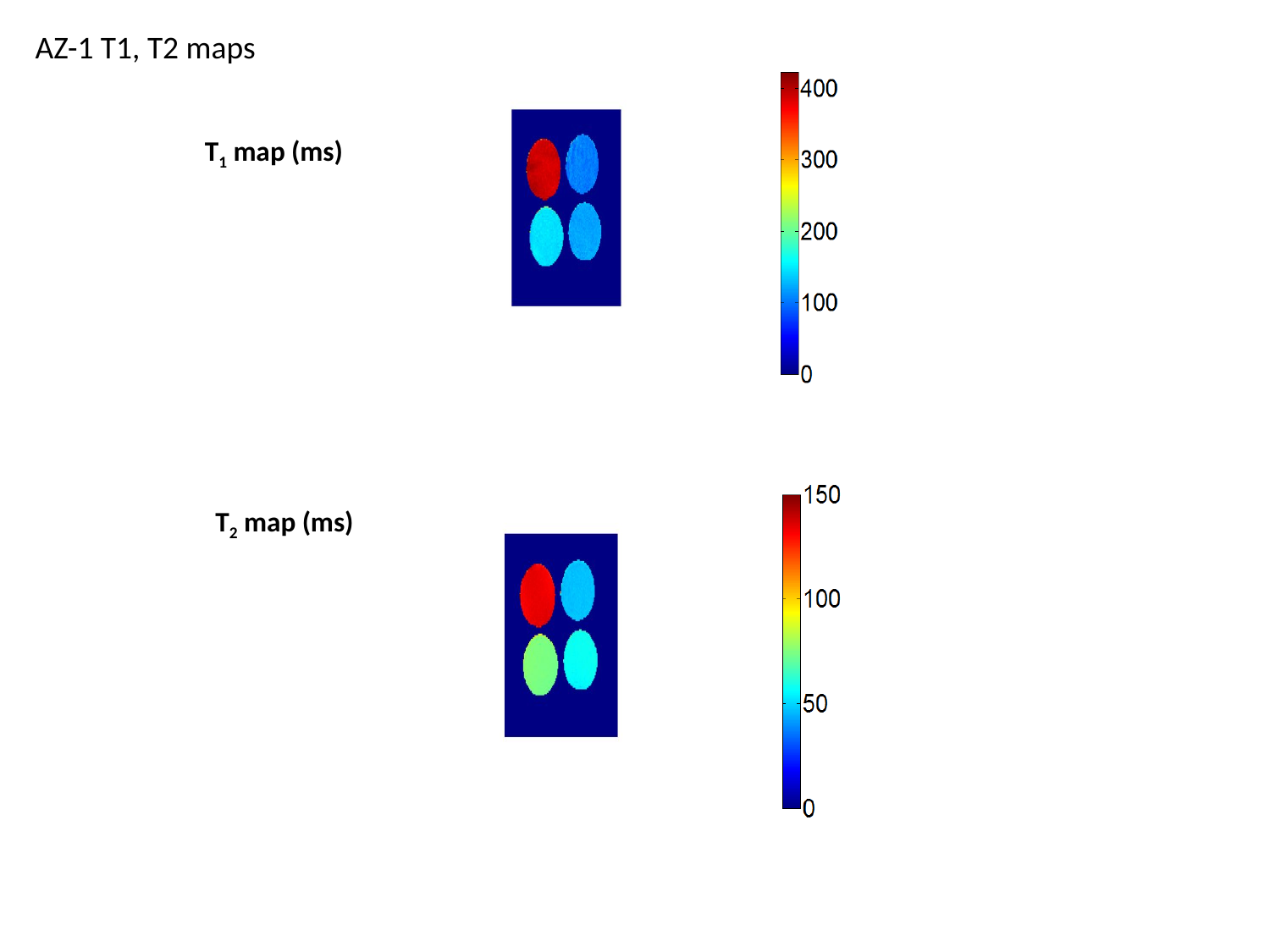

AZ-1 T1, T2 maps
A
D
T1 map (ms)
B
C
T2 map (ms)
A
D
B
C

## Slide 3
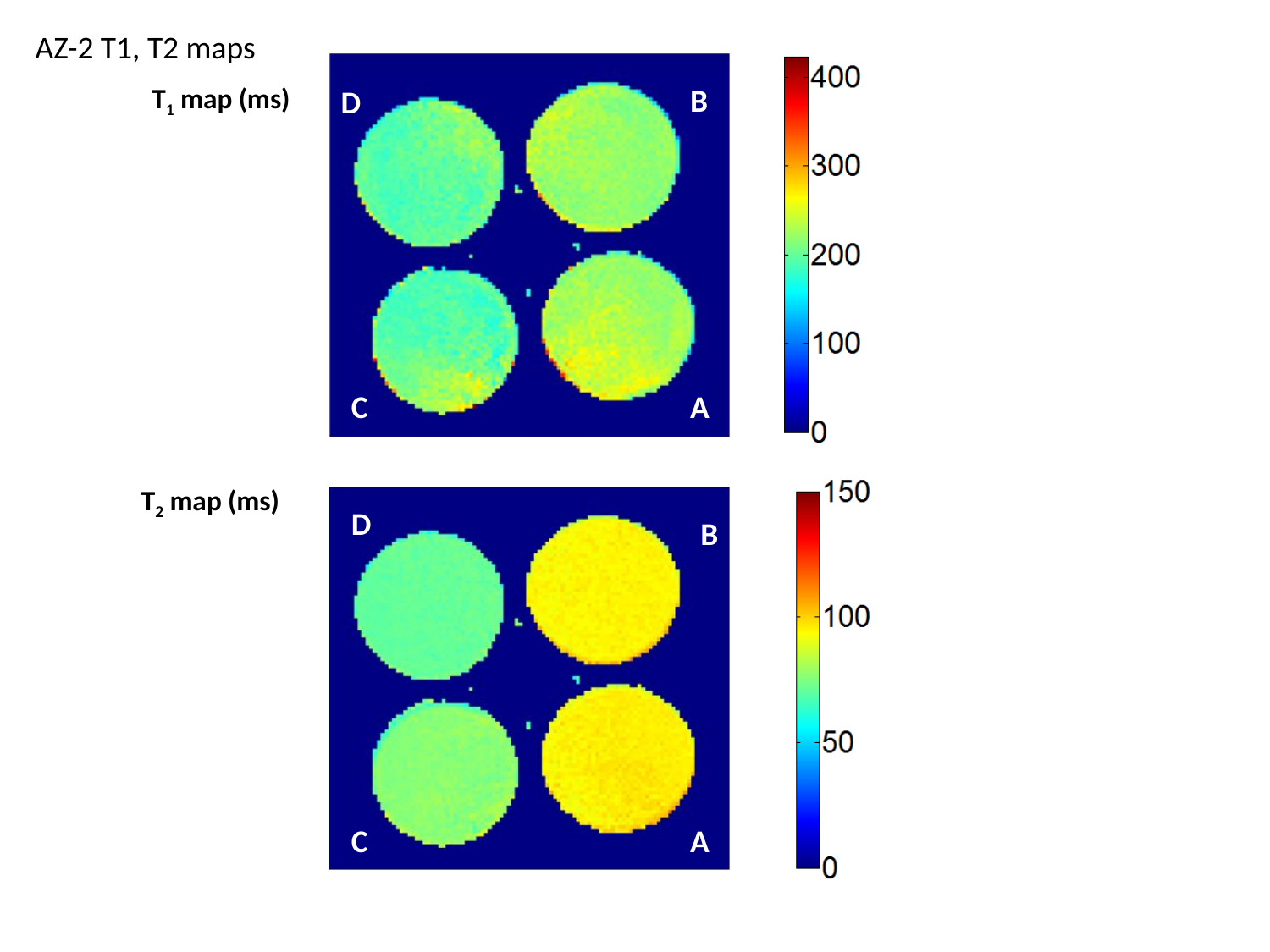

AZ-2 T1, T2 maps
T1 map (ms)
B
D
C
A
T2 map (ms)
D
B
C
A
C
A

## Slide 4
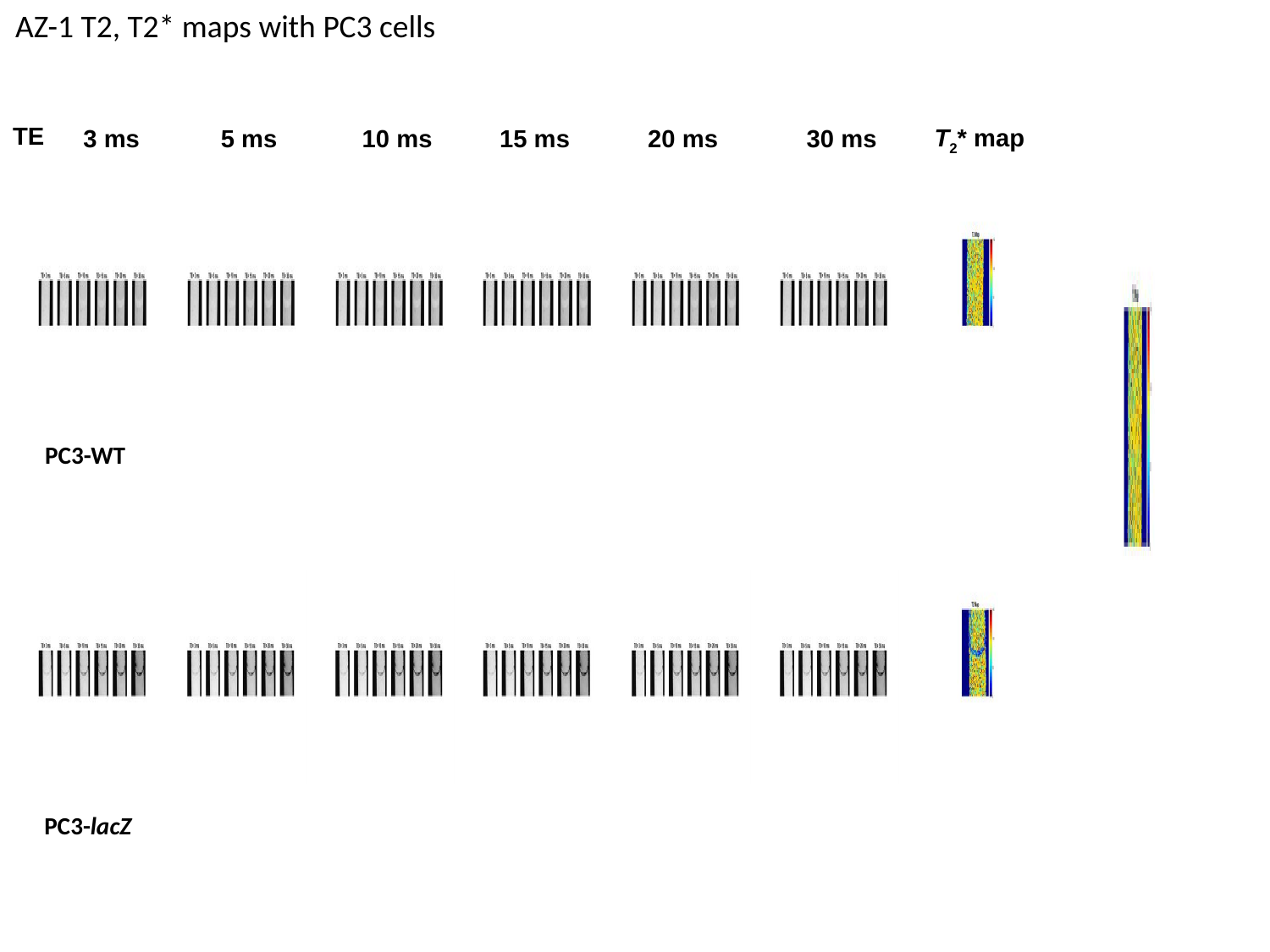

AZ-1 T2, T2* maps with PC3 cells
TE
T2* map
 3 ms
 5 ms
10 ms
15 ms
20 ms
30 ms
PC3-WT
PC3-lacZ

## Slide 5
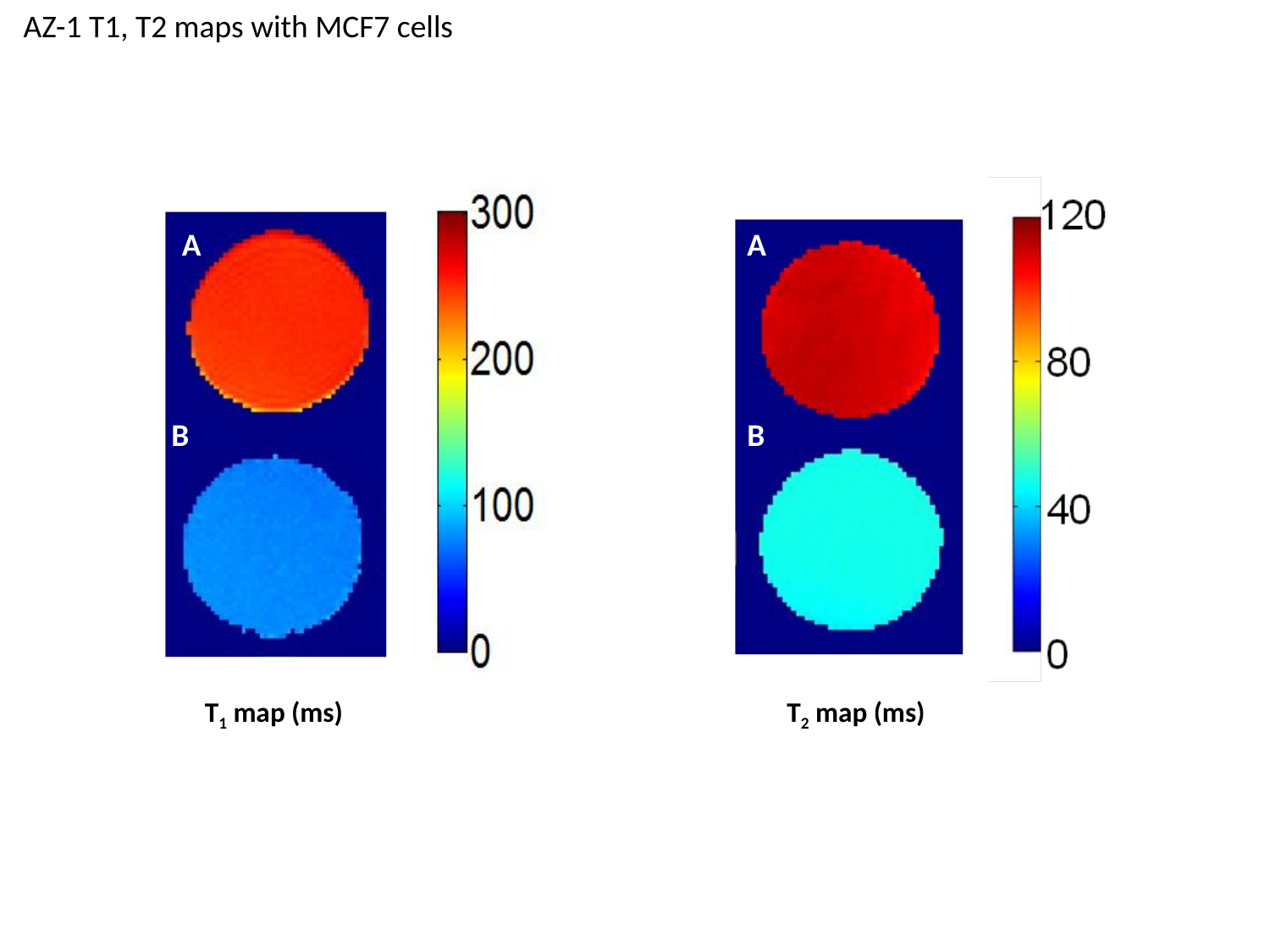

AZ-1 T1, T2 maps with MCF7 cells
A
A
B
B
T1 map (ms)
T2 map (ms)
B

## Slide 6
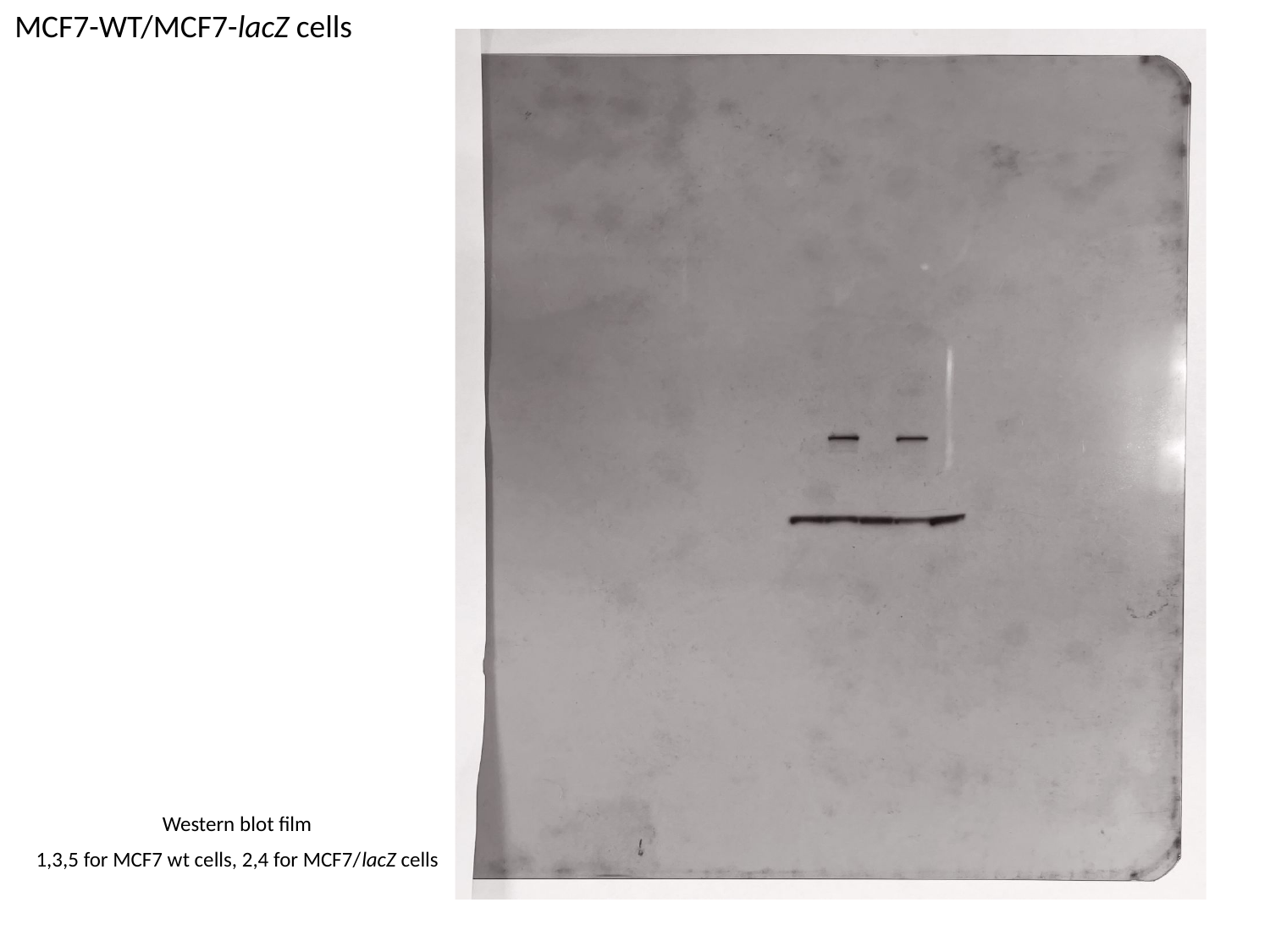

MCF7-WT/MCF7-lacZ cells
Western blot film
1,3,5 for MCF7 wt cells, 2,4 for MCF7/lacZ cells

## Slide 7
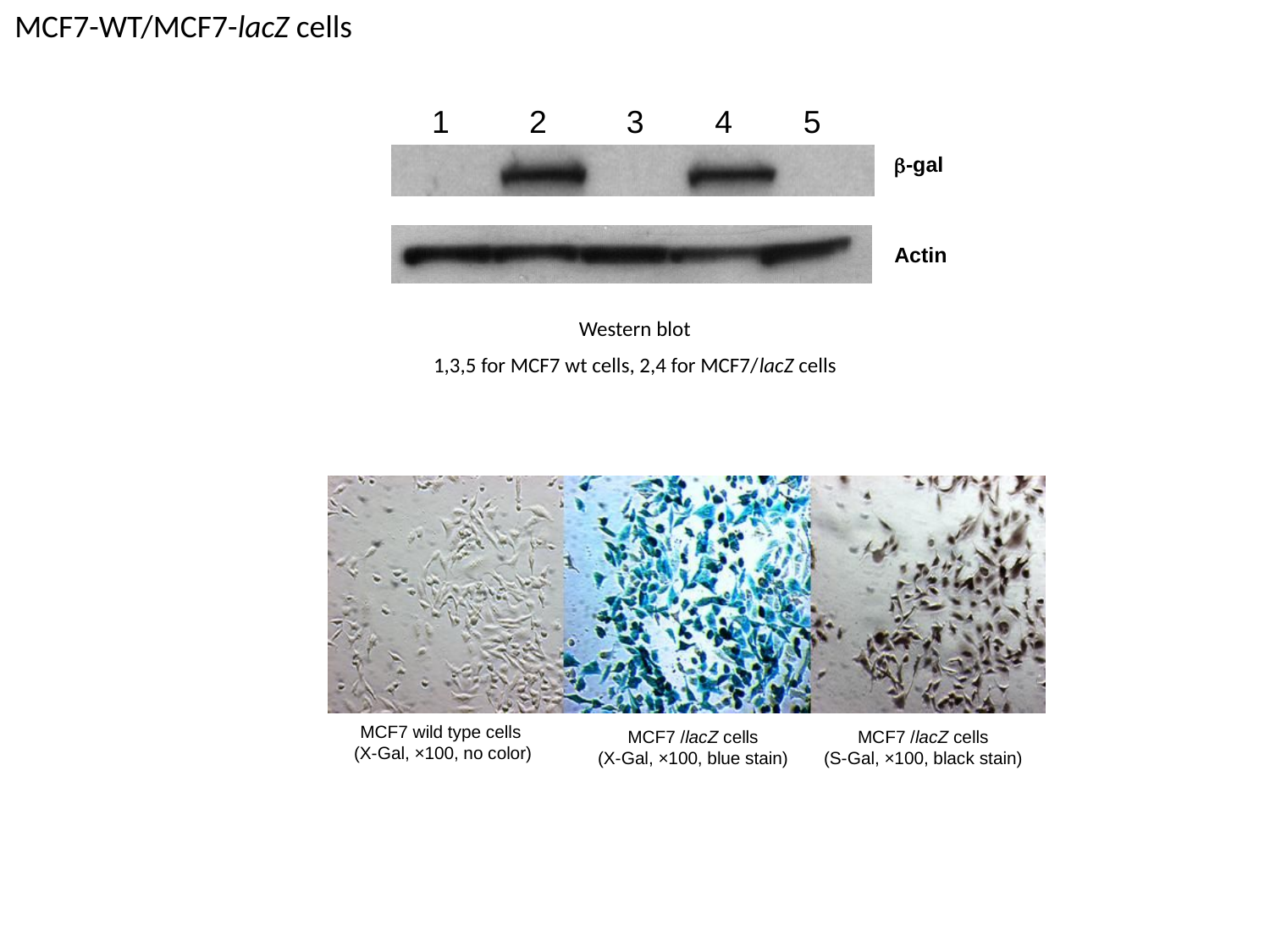

MCF7-WT/MCF7-lacZ cells
 1 2 3 4 5
β-gal
Actin
Western blot
1,3,5 for MCF7 wt cells, 2,4 for MCF7/lacZ cells
MCF7 wild type cells
(X-Gal, ×100, no color)
MCF7 /lacZ cells
(X-Gal, ×100, blue stain)
MCF7 /lacZ cells
(S-Gal, ×100, black stain)

## Slide 8
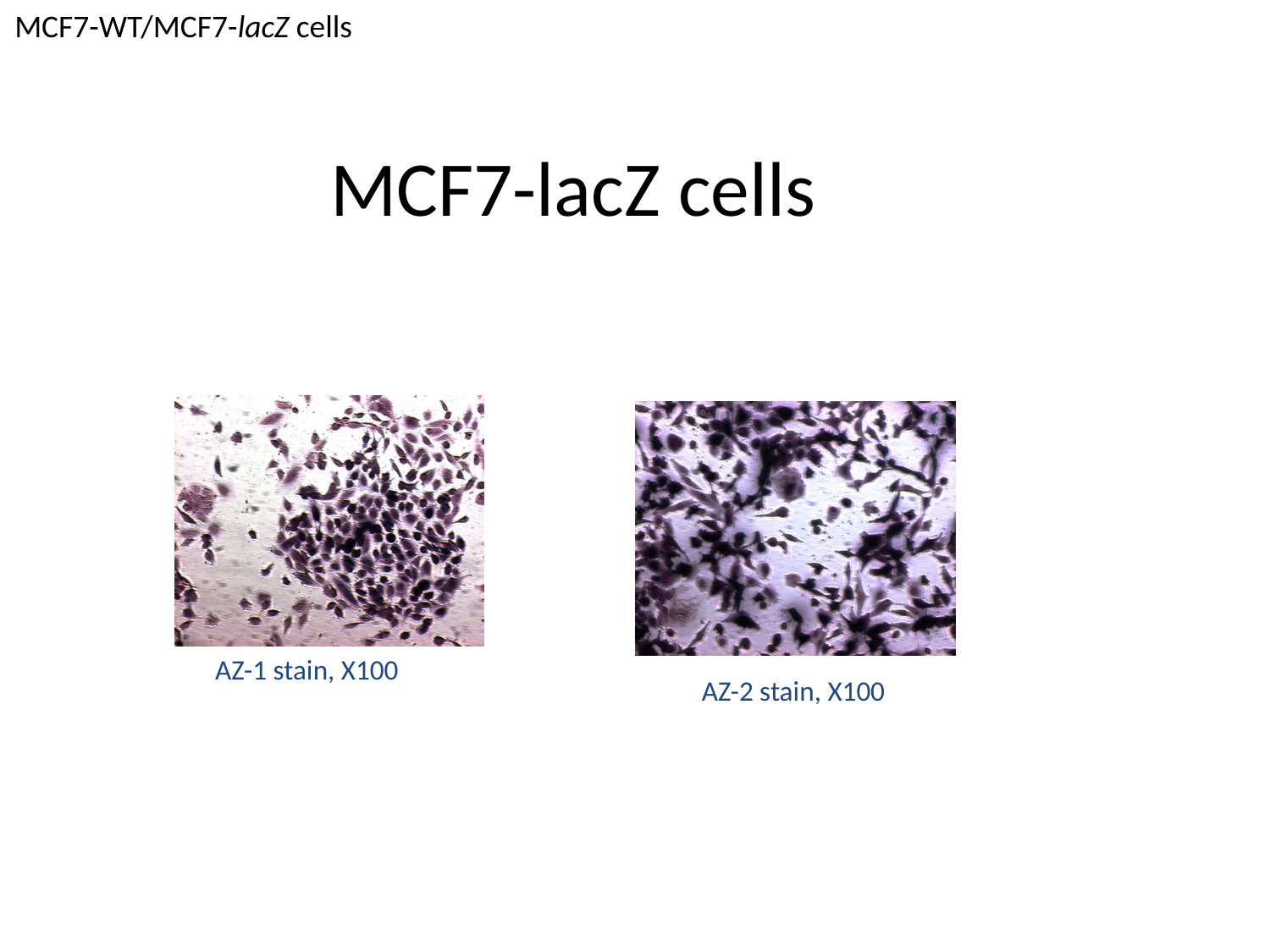

MCF7-WT/MCF7-lacZ cells
MCF7-lacZ cells
AZ-2 stain, X100
AZ-1 stain, X100

## Slide 9
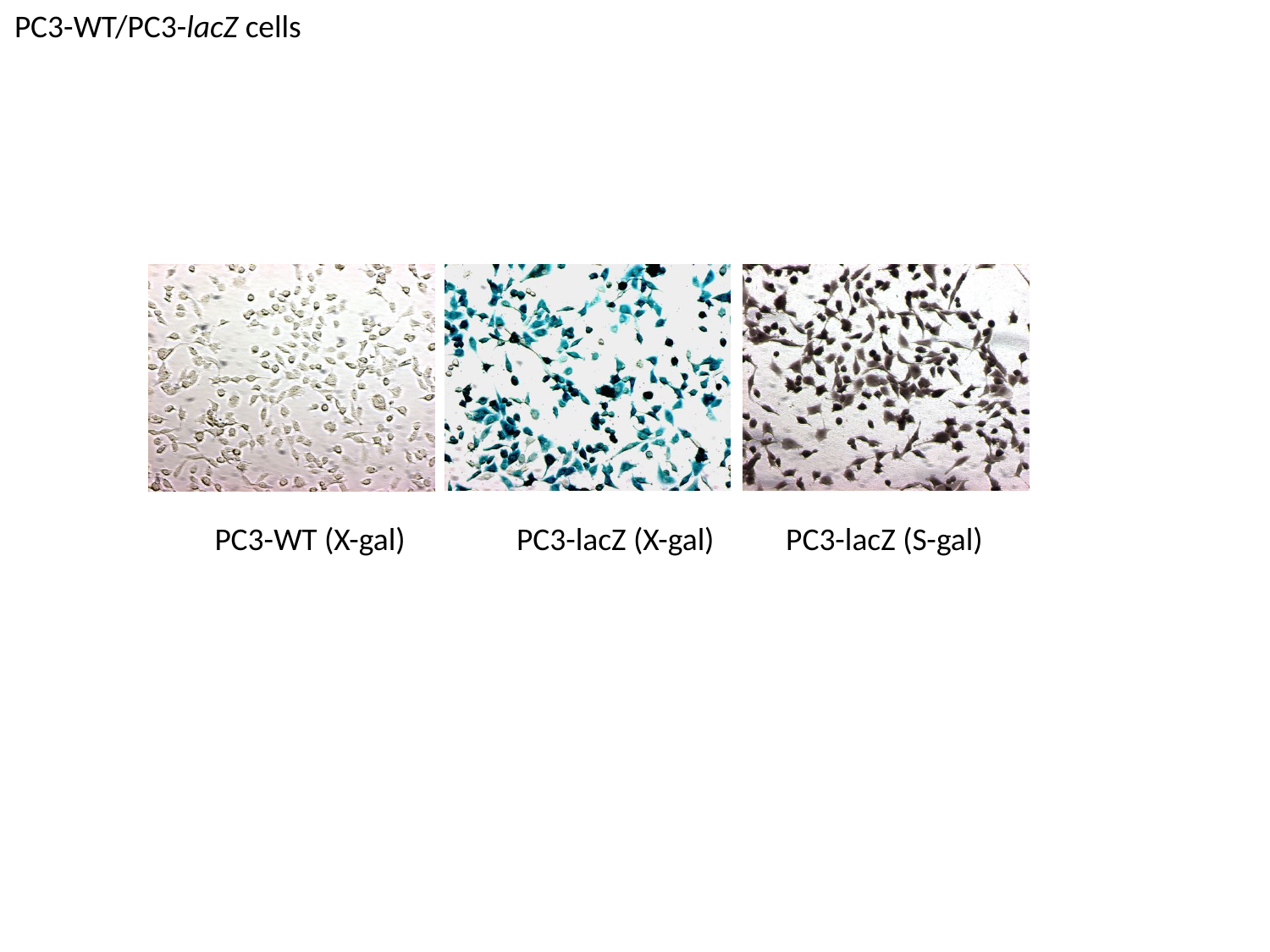

PC3-WT/PC3-lacZ cells
PC3-WT (X-gal)
 PC3-lacZ (X-gal) PC3-lacZ (S-gal)

## Slide 10
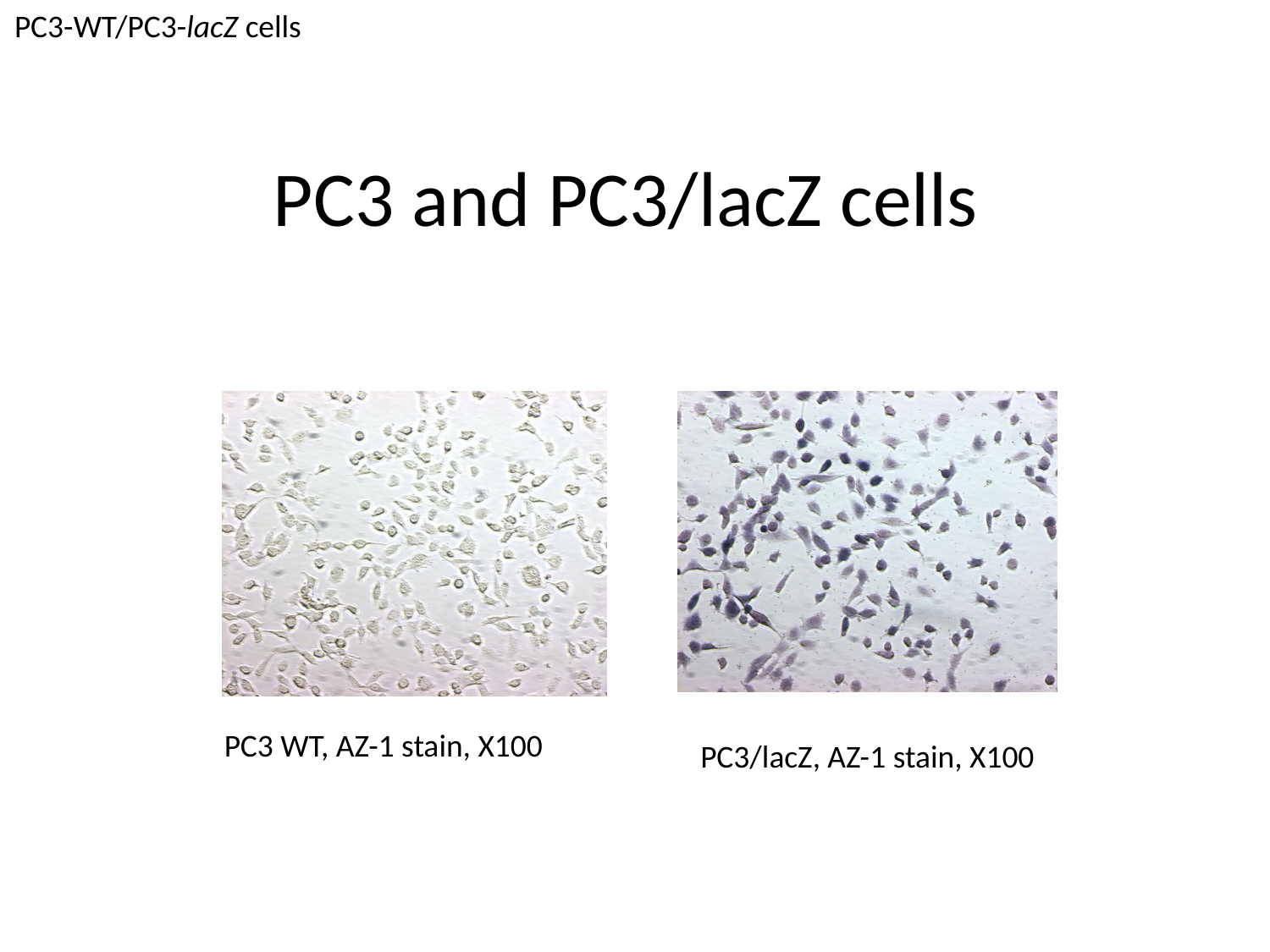

PC3-WT/PC3-lacZ cells
# PC3 and PC3/lacZ cells
PC3 WT, AZ-1 stain, X100
PC3/lacZ, AZ-1 stain, X100

## Slide 11
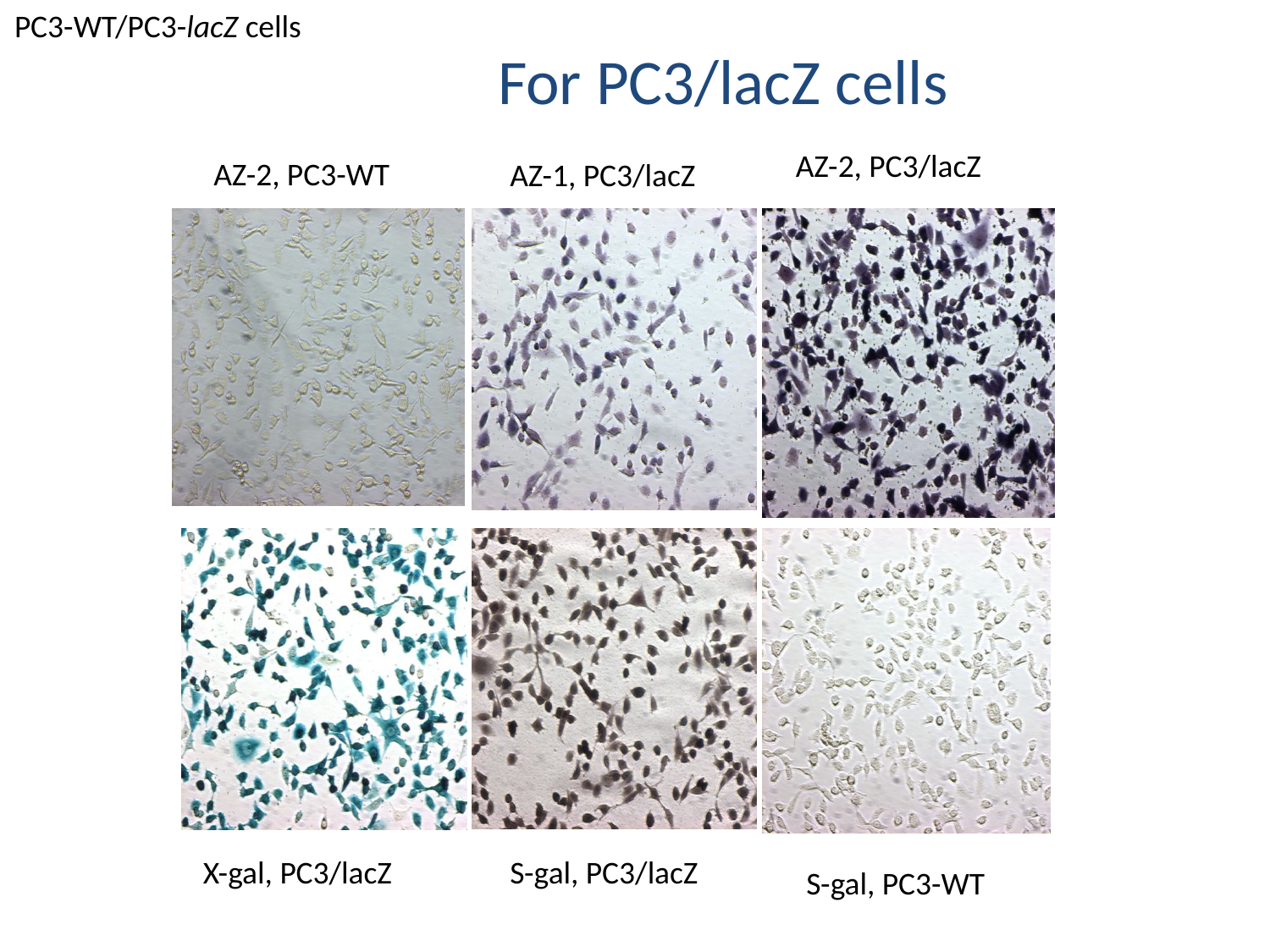

PC3-WT/PC3-lacZ cells
For PC3/lacZ cells
AZ-2, PC3/lacZ
AZ-2, PC3-WT
AZ-1, PC3/lacZ
X-gal, PC3/lacZ
S-gal, PC3/lacZ
S-gal, PC3-WT
